# ﻿Using genomics, morphometrics, and environmental niche modeling to test the validity of a narrow-range endemic snail, *Pateranantahala* (Gastropoda, Polygyridae)

**DOI:** 10.3897/zookeys.1158.94152

**Published:** 2023-04-20

**Authors:** Nathan V. Whelan, Ellen E. Strong, Nicholas S. Gladstone, Jason W. Mays

**Affiliations:** 1 Southeast Conservation Genetics Lab, Warm Springs Fish Technology Center, US Fish and Wildlife Service, 203 Swingle Hall, Auburn, Alabama, 36849, USA Auburn University Auburn United States of America; 2 School of Fisheries, Aquaculture, and Aquatic Sciences, College of Agriculture, Auburn University, 203 Swingle Hall, Auburn, Alabama, 36849, USA Warm Springs Fish Technology Center, US Fish and Wildlife Service Auburn United States of America; 3 Department of Invertebrate Zoology, National Museum of Natural History, Smithsonian Institution, PO Box 37012, MRC 163, Washington, DC 20013, USA National Museum of Natural History, Smithsonian Institution Washington United States of America; 4 Asheville Ecological Services Field Office, United States Fish and Wildlife Service, 160 Zillicoa ST, Asheville, NC 28801, USA Asheville Ecological Services Field Office, United States Fish and Wildlife Service Asheville United States of America

**Keywords:** 3RAD, generalized linear model, Maxent, morphology, Noonday Globe Snail, phylogenetic network, species tree, taxonomy

## Abstract

Terrestrial gastropods are among the most imperiled groups of organisms on Earth. Many species have a complex taxonomic history, often including poorly defined subspecies, most of which have not been the focus of modern systematics research. Genomic tools, geometric morphometrics, and environmental niche modeling were used to assess the taxonomic status of *Pateraclarkiinantahala* (Clench & Banks, 1932), a subspecies of high conservation concern with a restricted range of approximately 3.3 km^2^ in North Carolina, USA. A genome-scale dataset was generated that included individuals with morphologies matching *P.c.nantahala*, *P.c.clarkii*, and one individual with an intermediate form between *P.c.nantahala* and *P.c.clarkii* that was initially hypothesized as a potential hybrid. Mitochondrial phylogenetics, nuclear species tree inference, and phylogenetic networks were used to assess relationships and gene flow. Differences in shell shape via geometric morphometrics and whether the environmental niches of the two subspecies were significantly different were also examined. Molecular analyses indicated an absence of gene flow among lineages of *P.clarkii* sensu lato. Analyses rejected our hypothesis that the intermediate shelled form represented a hybrid, but instead indicated that it was a distinct lineage. Environmental niche models indicated significant differences in environmental niche between *P.c.clarkii* and *P.c.nantahala*, and geometric morphometrics indicated that *P.c.nantahala* had a significantly different shell shape. Given multiple lines of evidence, species-level recognition of *P.nantahala* is warranted.

## ﻿Introduction

Many conservation and environmental policies rely on functional units like species or subspecies (Primack 2014; Coates et al. 2018). For example, the U.S. Endangered Species Act defines species and subspecies as entities that can be listed as threatened or endangered. Therefore, applied conservation requires an informed taxonomy that accurately reflects diversity so conservation targets are not overlooked or overemphasized. In other words, modern systematics is essential for positive conservation outcomes.

Even though systematists debate the best approach for delineating species (Stankowski and Ravinet 2021), the definition of a species as a distinct evolutionary lineage is implicit in most species concepts (Mayden 1997; De Queiroz 2007). The taxonomic rank of subspecies, however, has been controversial. Unambiguous criteria for recognizing subspecies do not exist. Nevertheless, many systematists consider subspecies to be geographically distinct populations, often with distinct morphologies, that interbreed with other populations of the same species at contact zones (Patten 2015; Taylor et al. 2017). Under this definition, the defining characteristic of subspecies versus species is the ability of subspecies to routinely interbreed with other members of its species. Therefore, one would expect signatures of recent, or ongoing, gene flow between subspecies of the same species. If no such signature exists, then the two subspecies would be better considered as two distinct species.

The number of subspecies per species varies considerably among taxonomic groups. Generally, terrestrial snail groups exhibiting greater conchological complexity and larger ranges contain more subspecies (Páll-Gergely et al. 2019). This bias in use of subspecies implies that the number of subspecies may not always reflect actual terrestrial snail diversity. In the case of morphologically variable species with large ranges and discontinuous habitats, recognized subspecies may warrant species-level recognition. Given that few genome-scale studies have focused on terrestrial snails (but see Razkin et al. 2016; Phillips et al. 2020; Bober et al. 2021; Bamberger et al. 2022), the implicit hypothesis that gene flow occurs among subspecies has not been adequately tested in most cases.

**Figure 1.**
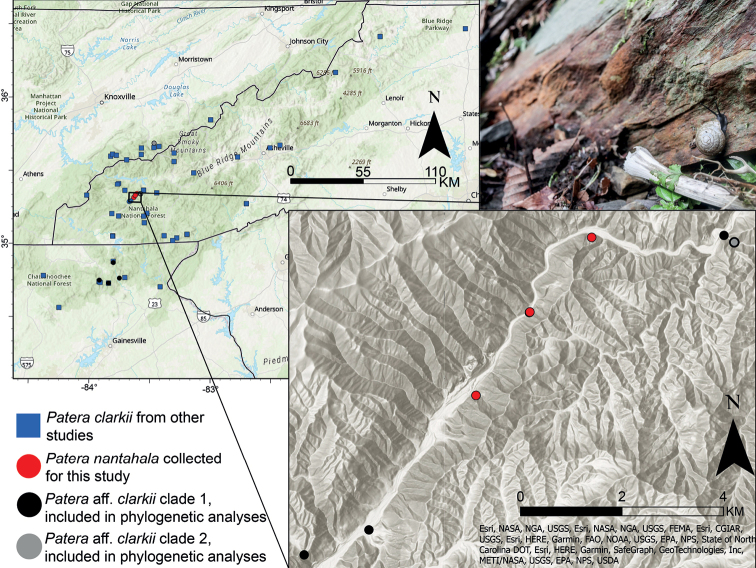
Map of records used for environmental niche modeling and phylogenetic analyses. Inset: Records collected here and included in molecular analyses. Only samples collected from locations in the inset were used for 3RAD analyses. Top right: photograph of *P.nantahala* in its natural habitat. Photograph by Gary Peeples (USFWS).

One terrestrial snail species that warrants closer scrutiny to assess the validity of subspecies and inform conservation is *Pateraclarkii* (I Lea, 1858). Currently, two subspecies are recognized: *Paterac.clarkii* and *Paterac.nantahala* (Clench & Banks, 1932), the latter of which is a federally listed subspecies under the U.S. Endangered Species Act (Greenwalt 1978). *Paterac.clarkii* is distributed in the southern Appalachian Mountains in northwestern Georgia, western North Carolina, and eastern Tennessee, USA (Fig. 1; Pilsbry 1940). *Paterac.nantahala*, the Noonday Globe Snail, inhabits a much smaller range, occupying approximately 3.3 km^2^ on the southeast slope, facing northwest, of the Nantahala Gorge in North Carolina (Fig. 1; Clench and Banks 1932; Van Devender 1984). Clench and Banks (1932) originally described *P.c.nantahala* as a distinct species in the genus *Polygyra* Say, 1818, but Pilsbry (1940) recognized *nantahala* at the rank of subspecies, within *Mesodonclarkii*, based on shell morphology. Emberton (1991) elevated *Patera* Albers, 1850 from *Mesodon* Férussac, 1821 and included *P.clarkii* in *Patera*. Emberton (1995) appeared to consider *P.c.nantahala* a valid subspecies when briefly discussing the listing status of polygyrids under the U.S. Endangered Species Act, but subspecies were not included in his list of species. Perez et al. (2014) was the first molecular phylogenetic study to infer that Patera species in the subgenus Patera, which includes *Pateraclarkii* (Emberton 1995), are monophyletic. However, no molecular study has assessed the status of *P.c.nantahala*.

**Figure 2.**
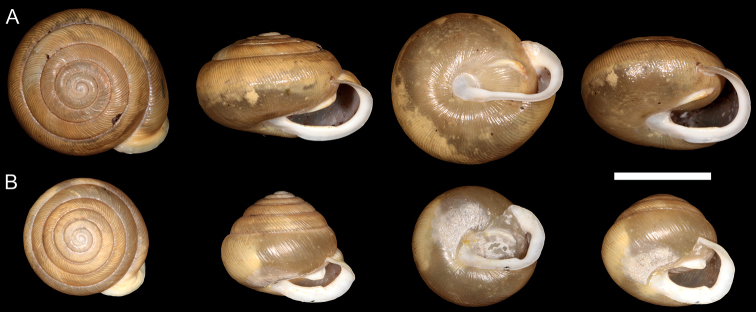
Photographs of type specimens **A** holotype of *Pateranantahala*, MCZ 86429 **B** syntype of *Pateraclarkii*, MCZ 93923. Scale bar: 1 cm.

Aside from morphological and range information from the original species description and museum records, little is known about *P.c.nantahala*. Based on the type specimens, *P.c.nantahala* has a larger shell diameter and a more depressed spire in relation to overall shell size (Fig. 2) than *P.c.clarkii*. *Paterac.nantahala* also has a smaller parietal tooth and a less pronounced denticle on the baso-palatal wall of the aperture. *Paterac.nantahala* inhabits heavily forested calcareous rocks that receive little daylight because of the Nantahala Gorge’s slope and position (Fig. 1), making its habitat unique from that of geographically proximate locations where *P.c.clarkii* is found. *Paterac.nantahala* was listed as threatened under the U.S. Endangered Species Act because of its extremely restricted range and concerns about potential habitat destruction from a proposed highway project (Greenwalt 1978). Currently, there are no plans to move forward with the highway project (Mays 2021), but *P.c.nantahala* relies on moist, shaded habitats that could be damaged by impacts to forest canopy such as wildfire, invasive species, and drought. For example, *P.c.nantahala* appeared to decrease in abundance after a prolonged drought in 2007–2009 (Mays 2021).

Objective morphological and phylogenetic analyses are needed to evaluate taxonomic hypotheses (Nekola and Horsák 2022). However, with so few data available, the taxonomic status of *P.c.nantahala* remains untested and its phylogenetic placement uncertain. Here, we generated mitochondrial and nuclear genomic datasets for *P.c.clarkii*, *P.c.nantahala*, an intermediate form, and the outgroup *P.perigrapta* (Pilsbry, 1894) to assess relationships among lineages and to test for evidence of gene flow. We also investigated morphological and environmental niche overlap between putative *P.clarkii* subspecies. Molecular results, in combination with habitat and morphological information, form the basis for proposed taxonomic revisions that better reflect diversity in *Patera* and will result in improved conservation focus.

## ﻿Materials and methods

### ﻿Taxon sampling and morphological documentation

**Figure 3.**
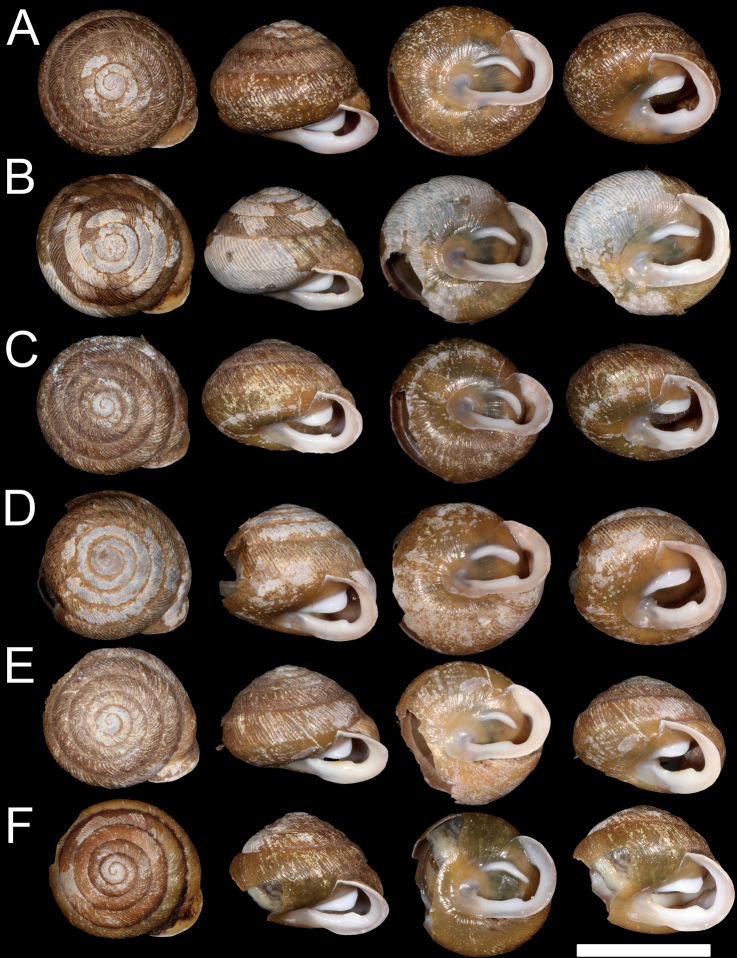
Shell morphology of Pateraaff.clarkii from Clade 1 **A**P.aff.clarkii 001, USNM 1522402 **B**P.aff.clarkii 002, USNM 1522403 **C**P.aff.clarkii 003, USNM 1522404 **D**P.aff.clarkii 005, USNM 1522406 **E**P.aff.clarkii 006, USNM 1522407 **F**P.aff.clarkii 007, USNM 1522408.

**Figure 4.**
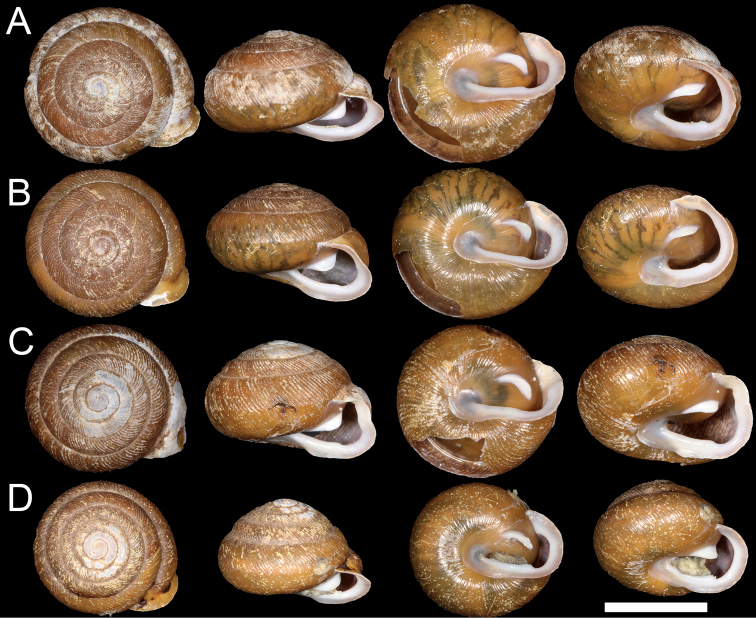
Shell morphology of *P.nantahala* and P.aff.clarkii from Clade 2 **A***P.nantahala* 001, USNM 1522409 **B***P.nantahala* 002, USNM 1522410 **C***P.nantahala* 003, USNM 1522411 **D**P.aff.clarkii 008, USNM 1522401.

*Paterac.clarkii*, *P.c.nantahala*, and *P.perigrapta* were collected from eastern North Carolina in the Nantahala National Forest (Table 1; Figs 1, 3, 4). Collections included one individual with a morphology intermediate between the type specimens of *P.c.clarkii* and *P.c.nantahala* that we initially hypothesized was a hybrid between the two putative subspecies (Fig. 4D; individual “P.aff.clarkii 008”). Sampling locations were chosen strategically as likely contact zones, making the taxon sampling of this study well-suited to test the taxonomic status of the two subspecies. Individuals were placed in 95% ethanol in the field. A ~ 3 mm^3^ tissue clip was taken from each individual for DNA extraction, and some shells had to be cracked to access the tissue. All shells were photographed. The shell vouchers for all sequenced individuals have been deposited at the National Museum of Natural History (Table 1).

**Table 1.** Collection localities, molecular data accession numbers, and museum catalog numbers of individuals collected in this study.

Individual	Collection Location	GPS Coordinates	USNM ###	GenBank ## (COI, H3, 28S)	SRA ##
Pateraaff.clarkii 001	Winding Stairs next to Queens Creek	35.285, -83.668	1522402	OQ617117, OQ628057, OQ628452	SRX19664328
Pateraaff.clarkii 002	Winding Stairs next to Queens Creek	35.285, -83.668	1522403	OQ617115, OQ628064, OQ628453	SRX19664327
Pateraaff.clarkii 003	Winding Stairs next to Queens Creek	35.285, -83.668	1522404	OQ617116, OQ628063, OQ628454	SRX19664326
Pateraaff.clarkii 004	Adjacent to Wesser Creek and Nantahala River	35.334, -83.654	1522405	OQ617118, OQ628065, OQ628455	SRX19664325
Pateraaff.clarkii 005	Adjacent to Handpole Branch	35.281, -83.682	1522406	OQ617119, OQ628058, OQ628456	SRX19664324
Pateraaff.clarkii 006	Adjacent to Handpole Branch	35.281, -83.682	1522407	–––––––––, OQ628059, OQ628457	SRX19664323
Pateraaff.clarkii 007	Adjacent to Handpole Branch	35.281, -83.682	1522408	OQ617120, OQ628060, OQ628458	SRX19664322
*Pateranantahala* 001	Southeast Cliff of Nantahala Gorge	35.308, -83.644	1522409	OQ617122, OQ628062, OQ628460	SRX19664333
*Pateranantahala* 002	Southeast Cliff of Nantahala Gorge	35.308, -83.644	1522410	OQ617123, OQ628056, OQ628461	SRX19664332
*Pateranantahala* 003	Northeast corner of Nantahala Gorge	35.336, -83.620	1522411	OQ617124, OQ628055, OQ628462	SRX19664329
*Pateraperigrapta* 001	Winding Stairs next to Queens Creek	35.285, -83.668	1522398	OQ617112, OQ628052, OQ628463	SRX19664334
*Pateraperigrapta* 002	Adjacent to Wesser Creek	35.333, -83.587	1522399	OQ617114, OQ628053, OQ628464	SRX19664330
*Pateraperigrapta* 003	Wayah Road, Nantahala	35.257, -83.656	1522400	OQ617113, OQ628054, OQ628465	–––––––––––
Pateraaff.clarkii 008	Adjacent to Wesser Creek	35.333, -83.587	1522401	OQ617121, OQ628061, OQ628459	SRX19664331

We also obtained loans of type material and other *Pateraclarkii* ssp. lots from three major natural history collections: Harvard Museum of Comparative Zoology, the Academy of Natural Sciences Philadelphia, and the National Museum of Natural History (Suppl. material 1). Subspecies identification was based on collector identification, location of collection, and comparisons to type material. Institutional abbreviations used in the text are:

**MCZ**Harvard Museum of Comparative Zoology;

**ANSP**Academy of Natural Sciences Philadelphia;

**USNM**National Museum of Natural History.

For mitochondrial analyses (see below), we obtained sequences of *Patera* and other Polygyridae from Perez et al. (2014: fig. 1). Sequences were obtained directly from the authors as the data were not available on GenBank. No other sequences for *Patera* were publicly available at the time of this study.

### ﻿Genetic data generation

DNA was extracted from tissue clips with the Qiagen DNeasy Plant Mini Kit using a slight modification to incorporate a proteinase K tissue digestion step. A plant kit was used because it handles mucopolysaccharides in snail tissue better than standard animal kits (Whelan et al. 2019). DNA was quantified on a Qubit fluorometer. An aliquot was taken from each DNA extraction and diluted to 20 ng/µL.

Three genes were targeted for Sanger sequencing: 1) mitochondrial cytochrome *c* oxidase I, 2) nuclear 28S rRNA, and 3) nuclear Histone H3. PCR amplification for COI used primers dgLCO-1490 (5’ GGTCAACAAATCATAAAGAYATYGG 3’) and dgHCO-2198 (5’TAAACTTCAGGGTGACCAAARAAYCA 3’) (Meyer 2003). Reactions occurred in 25 µL volumes consisting of 5 µL 5× GoTaq Flexi Buffer (Promega), 2.5 µL MgCl_2_ (25 mM), 1 µL of each primer (10 µM), 1 µL dNTP solution (10 mM), 0.1 U GoTaq DNA polymerase (Promega), and 20 ng whole genomic DNA. PCR cycling used an initial denaturation at 94 °C for 2 min; 35 cycles of 94 °C for 30 s, 45 °C for 30 s, 72 °C for 1 min; and a final extension at 72 °C for 5 mins. PCR amplification for 28S used primers 28S-VI (5’ AAGGTAGCCAAATGCCTCATC-3’) and 28S-X (5’-GTGAATTCTGCTTCATCAATGTAGGAAGAGCC-3’) (Hillis and Dixon 1991). Reactions occurred in 25 µL volumes consisting of 5 µL GoTaq Flexi Buffer, 2.5 µL MgCl_2_ (25 mM), 1 µL each primer (10 µM), 1 µL dNTPs (10 µM), 0.1 U GoTaq DNA polymerase, and 10 ng genomic DNA. PCR for 28S cycling used an initial denaturation at 94 °C for 2 min; 30 cycles of 94 °C for 30 s, 50 °C for 30 s, 72 °C for 30 s; and a final extension at 72 °C for 5 mins. PCR for H3 used primers H3F (5’-ATGGCTCGTACCAAGCAGACVGC-3’) and H3R (5’-ATATCCTTRGGCATR ATRGTGAC-3’) (Colgan et al. 1999), and the same reaction chemistry as 28S. H3 PCR cycling used initial denaturation at 94 °C for 2 min; 30 cycles of 94 °C for 30 s, 55 °C for 30 s, 72 °C for 30 s; and a final extension at 72 °C for 5 mins. Raw PCR products were purified using the New England Biolabs Monarch PCR & DNA cleanup kit following manufacturer’s protocol. Cleaned PCR products were sent to GeneWiz for Sanger sequencing in both directions using the same primers as used in PCR reactions.

After finding a lack of variation in nuclear genes (see results), we generated a genome-scale dataset for two individuals of *P.perigrapta* and all *P.clarkii* sensu lato (s.l.) that we successfully Sanger sequenced. To do this, we used the “3RAD” restriction site associated DNA sequencing reduced representation sequencing approach (RAD-seq; Bayona-Vásquez et al. 2019). 3RAD has advantages over other RAD-seq approaches by reducing adapter-dimer formation, allowing incorporation of a random 8 bp Illumina i5 index for removing PCR duplicates during assembly, and using a sequencing strategy (2 × 150 paired-end) that results in 200 bp, or greater, contigs. The long contigs generated with 3RAD, compared to some other RADseq approaches (e.g., 2bRAD; Wang et al. 2012), are particularly useful for phylogenetics. We followed the original 3RAD protocol with slight modification (full protocol available from https://github.com/NathanWhelan/3RAD_protocols/). The digestion step used restriction enzymes Nhel, EcoRI, and XbaI. *Patera* libraries were combined with samples from other studies that had unique barcodes, resulting in 192 libraries that were sequenced at the University of Oregon Genomics and Cell Characterization Core facility on an Illumina NovaSeq 6000 using an SP flow cell with 2 × 150 paired-end sequencing chemistry.

### ﻿Molecular data analyses

Raw Sanger sequencing chromatograms were visualized in Geneious Prime and checked for sequencing errors. For the two nuclear genes, sites with two chromatogram peaks of equal intensity on both the forward and reverse sequences were coded as heterozygous using standard IUPAC codes. Each gene was aligned with Clustal Omega 1.2.2 (Sievers et al. 2011). All 28S sequences were identical, so 28S was not used in phylogenetic analyses. We inferred a COI mitochondrial gene tree and an H3 nuclear gene tree separately. First, the best-fit substitution models and partitions were identified with ModelFinder using the Bayesian information criterion (BIC) (Kalyaanamoorthy et al. 2017) as implemented in IQTREE 1.6.12 (Nguyen et al. 2015); codon positions were used as starting blocks. Maximum likelihood tree inference was then done in IQTREE using best-fit models and partitions. Tree search used default parameters, except perturbation strength was set to 0.2 and number of unsuccessful steps to stop tree inference was set to 500. Support was measured with 1,000 ultrafast bootstrap replicates (Hoang et al. 2018). Average pairwise distances among *P.clarkii* s.l. clades were calculated in MEGAX 10.2.6 (Kumar et al. 2019).

An automatic species delimitation approach was used to generate species-level taxonomic hypotheses. For this, we used COI data with Assemble Species by Automatic Partitioning (ASAP; Puillandre et al. 2021). ASAP has improved performance and less subjectivity in choosing delimitation schemes than its predecessor, the widely used Automatic Barcode Gap Discovery method (ABGD; Puillandre et al. 2012). ASAP was chosen over other methods because ABGD was previously shown to work well compared to other methods on gastropods with low dispersal ability (Strong and Whelan 2019). We also chose to use ASAP because it is not based on the coalescent model, and methods that use the coalescent tend to over split species (Sukumaran and Knowles 2017; Strong and Whelan 2019). For ASAP, the COI dataset was trimmed of outgroups to only include *P.c.clarkii* and *P.c.nantahala*. First, the best fit model for the trimmed dataset was inferred with ModelFinder in IQ-TREE. Second, we calculated best-fit model maximum likelihood distances among individuals with PAUP* 4.0a build 169 (https://paup.phylosolutions.com) using model parameters inferred by ModelFinder. Finally, ASAP analyses were performed with the ASAP web server (https://bioinfo.mnhn.fr/abi/public/asap/) using maximum likelihood distances.

Raw 3RAD sequence data were demultiplexed into individual libraries with the STACKS 2.53 script process_radtags (Rochette et al. 2019). One mismatch per barcode was allowed. Reads that lacked restriction enzyme sites were discarded. After demultiplexing, PCR clones in each library were removed using the STACKS script clone_filter. PCR clones were identified with the random sequence i5 index used during library preparation.

After demultiplexing and clone filtering, data were assembled using the STACKS denovo_map.pl pipeline. We first used the method described by Paris et al. (2017) to identify appropriate assembly parameters. The best parameters for our data were determined to be a minimum stack depth of three (-m 3), five mismatches allowed between stacks within individuals (-M 5), and five mismatches allowed between stacks among individuals (-n 5). Contigs were assembled by denovo_map using paired-end information. All other assembly parameters were set to default. After assembly, the STACKS program populations was used for final data filtering. To pass filters, loci had to be present in 100% of individuals, have a minimum minor allele frequency of 0.025, and have an observed heterozygosity frequency of no more than 0.5. These parameters were chosen to eliminate missing data and filter potential paralogs or sequencing errors. All SNPs per locus were retained. Processed data were output into various file formats by STACKS for downstream analyses.

Some contigs, or RAD loci, did not have overlapping reads because the locus was longer than 300 bp, which STACKS represented as a string of Ns. These were removed prior to phylogenetic analyses with the custom script noGaps-nucleotides.sh. Maximum likelihood gene trees were inferred for each RAD locus with IQTREE. ModelFinder, as implemented in IQTREE, was used for substitution testing using the BIC; partition finding was not done because RAD loci are unlikely to be found only in exons. Tree inference and bootstrapping for RAD loci were the same as for Sanger sequenced genes.

ASTRAL III (Zhang et al. 2018) and the RAD-loci nuclear gene trees were used to infer a species tree. This method uses the multispecies coalescent to resolve gene tree conflict and assumes that all gene tree discordance is a result of incomplete lineage sorting (Rannala and Yang 2003). Prior to using the inferred maximum likelihood trees of each gene for species tree inference, all branches with 10% ultrafast bootstrap support or less were collapsed with Newick Utilities (Junier and Zdobnov 2010). Collapsed maximum likelihood trees for each gene and default parameters were used as ASTRAL input. Individuals were not assigned a priori taxon designations. Support was measured with local posterior probability (Sayyari and Mirarab 2016).

Given that focal taxa were putative subspecies where some gene flow is expected, introgression is a potential cause of gene tree discordance (Maddison 1997). To test for a signal of introgression, we used the phylogenetic network method SNAQ (Solís-Lemus and Ané 2016) implemented in PhyloNetworks (Solís-Lemus et al. 2017). Unlike implicit network approaches that visualize discordance (e.g., SplitsTree; Huson and Bryant 2006), networks inferred with SNAQ can represent explicit reticulation events and all nodes represent ancestors (Solís-Lemus and Ané 2016). SNAQ is also an appropriate method for the current taxon sampling as genome-wide markers, combined with a network approach, allows estimating gene flow across the evolutionary history of a lineage. Maximum likelihood trees used for ASTRAL input were used in SNAQ to calculate concordance factors, and the ASTRAL species tree was used as the starting tree. Five separate networks that allowed for 0–4 reticulations (*h*), respectively, were inferred with ten replicates each. The best-fitting number of reticulations was assessed by examining the log pseudolikelihood profile of *h*, following Solís-Lemus and Ané (2016). Inferred networks that conflicted with the outgroup position of *P.perigrapta* were discarded, and we instead retained the network for each *h* with the highest log pseudolikelihood value that did not conflict with root position. Goodness-of-fit of each network was also examined by plotting observed concordance factors versus expected concordance factors for each network. The R package ggplot2 (Wickham 2009) was used for plotting.

### ﻿Morphological analyses

**Figure 5.**
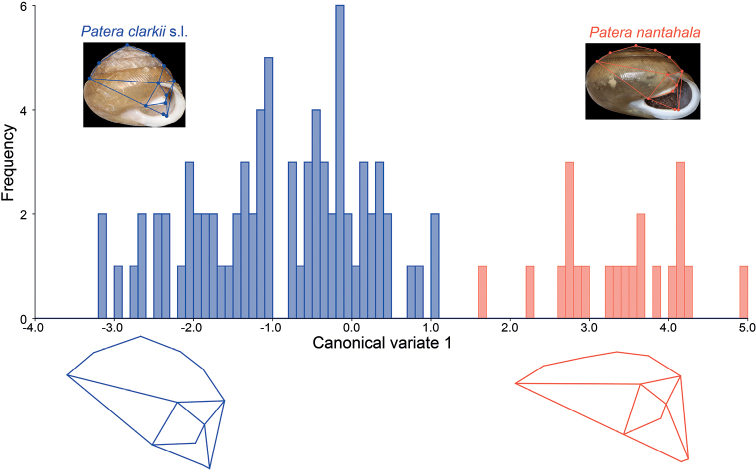
Landmarks used for geometric morphometrics and history of canonical variate scores. Shells are type specimens and points represent landmarks connected by wireframe that shows shape variation. Wireframe graphs under CVA plots represent extremes and show shape changes associated with canonical variates.

All shell vouchers for molecular samples and most shells obtained from museum collections were used to assess morphological similarity between *P.c.nantahala* and *P.c.clarkii* via geometric morphometrics (Suppl. material 1). We used a maximum of four shells per museum lot; shells with damage in areas important for assigning landmarks were also excluded. Shells were photographed in apertural view with the axis of coiling parallel to the camera sensor (Fig. 5). Photographs were taken on a Canon E0S 80D with a 100 mm f/2.8 macro lens. Photographs of a ruler at the same scale as the shell photographs were also taken so we could test differences in shell size in addition to shape.

We used tpsUtil version 1.82 (Rohlf 2021a) to convert photographs to tps file format. Photographs were reordered randomly to limit landmark placement biases systematically affecting samples from the same lot. tpsDig2 version 2.32 (Rohlf 2021b) was used to place 12 landmarks on each shell (Fig. 5). Landmarks were chosen based on inferred ability to consistently place them in homologous positions.

Geometric morphometric analyses were conducted in MorphoJ (Klingenberg 2011). First, a Procrustes fit was applied to the dataset to account for differences in shell size, position, and image rotation. Correct landmark digitization and the presence of outliers were checked by eye for all samples using Mahalanobis distance (Klingenberg and Monteiro 2005), which was inferred by MorphoJ to be more appropriate than Squared Procrustes distance for our dataset. Incorrect digitization of landmarks was corrected via the “Swap Landmark” command when the deviation from the average of any two landmarks on the same shell clearly pointed at each other (see MorphoJ manual for more details).

Differences in shape were measured using two statistical tests. First, a Procrustes ANOVA was performed to test for significant differences in shape between the two putative subspecies. Then, a canonical variate analysis (CVA) was performed in MorphoJ to visualize shape differences and further assess evidence for shape differences between *P.c.clarkii* and *P.c.nantahala*. For the CVA, a permutation test of pairwise distances between putative subspecies was performed to test for significance using 1,000 iterations per comparison. Wireframe graphs were plotted to visualize morphological variation along the CVA axis. A Procrustes ANOVA was also performed to test for significant differences in centroid size, which is a measure of shell size.

### ﻿Environmental niche modeling

*Paterac.nantahala* is an ideal taxon for examining the utility and accuracy of environmental niche models because its range is extremely restricted and well defined. We also wanted to quantify potential environmental niche overlap between *P.c.clarkii* and *P.c.nantahala*. First, we downloaded collection records of *P.clarkii* from the Global Biodiversity Information Facility (GBIF) that had latitude and longitude information (GBIF.org 2022). One record of *P.c.clarkii* from New Jersey was removed from the downloaded dataset (GBIF.org 2022) as *P.clarkii* is not known to occur north of North Carolina (Hubricht 1985). A GBIF record of *P.c.nantahala* from iNaturalist was also removed because the precise location was obscured reflecting the species’ threatened status. Records from Perez et al. (2014) and our own collections were added to those downloaded from GBIF (Fig. 1). Perez et al. (2014) did not provide latitude and longitude, so we determined reported locations based on their descriptions and coordinates determined from Google Earth. To reduce potential biases associated with spatial autocorrelation of species records, we spatially rarefied locality records at a distance of 1 km with SDMtoolbox 2.0 (Brown et al. 2017) in ESRI ArcGIS Pro; 1 km was chosen given the small distance between *P.c.nantahala* records.

Continuous environmental variables that covered the spatial extent of collection records (Fig. 1) were downloaded from publicly available sources as raster files. Bioclimatic data from WorldClim (Fick and Hijmans 2017) were downloaded at 30 second resolution with the R package raster (Hijmans 2022). Erodibility and albedo raster files were downloaded from the USA Soils dataset (SSURGO; Soil Survey Staff 2022) via ESRI ArcGIS Living Atlas of the World at 30-meter resolution. Categorical soil map unit data were also downloaded from SSURGO to examine differences in habitat, but categorical data were not included in environmental niche models. Forest canopy cover (i.e., proportion of floor covered by vertical projection of tree crowns), forest canopy base height (i.e., average height to the top of tree canopy), vegetation height (i.e., vertically projected cover of live plants), and vegetation cover (i.e., average height of dominant vegetation) were downloaded at 30-meter resolution from LANDFIRE version LF 2016 Remap (LANDFIRE 2020). Elevation data were downloaded from The National Map at 1/3 arc-second resolution (U.S. Geological Survey 2020); elevation data at 1/3 arc-second resolution were only available as multiple raster files across the study extent, so raster files were combined in ESRI ArcGIS Pro using the “Mosaic to Layer” tool. Slope and aspect data were created from the elevation data in ArcGIS Pro using the “Slope” and “Aspect” tools, respectively. Environmental data used in niche modeling were chosen to represent potentially unique features of the Nantahala Gorge and its habitat (e.g., steep slope, soil type, vegetation, and limited sunlight).

Environmental data raster files were trimmed to cover the area where samples were collected (Fig. 1) in ArcGIS Pro and bicubic resampling was used to ensure each raster had the same cell size of 0.0005. Data were resampled to a cell size of 0.0005 to balance processing speed and resolution of taxa whose records were sometimes barely more than 1 km apart. For data with the USGS version of USA Contiguous Albers Equal Area Conic as their native projection, we used ArcGIS pro to reproject to the World Geodetic System 1984 projection. After data transformation, all data raster files were exported from ArcGIS Pro as .tif files for use in R. Data points of “NA” were changed to “0” because some datasets (e.g., LANDFIRE) coded water bodies as NA, rather than 0.

Raster files were loaded into R with the “raster” command of the package raster and stacked into a single variable. Correlation of the different environmental data layers was assessed on *P.c.clarkii* collection records with “raster.cor.matrix” and “raster.cor.plot” commands of the R package ENMTools (Warren et al. 2021). Correlated variables were determined with only the *P.c.clarkii* dataset because there were considerably more records for *P.c.clarkii* than *P.c.nantahala*. We removed all but one of any given environmental layer that had high correlation with other layers (Pearson correlation coefficient > 0.70; see Table 2 for variables used in final datasets). To examine effects of performing niche modeling using bioclimatic data such as temperature and precipitation versus geographical and biotic data such as elevation and vegetation cover, we created three datasets: 1) all variables; 2) only bioclimatic data; 3) biotic, geological, and geographic (Table 2).

**Table 2.** Data used for environmental niche modeling.

Data Source	Environmental Layer	Data type	Characteristics
WorldClim	BIO1	Bioclimatic	Annual mean temperature
WorldClim	BIO2	Bioclimatic	Mean diurnal range
WorldClim	BIO3	Bioclimatic	Isothermality
WorldClim	BIO4	Bioclimatic	Temperature seasonality
WorldClim	BIO7	Bioclimatic	Temperature annual range
WorldClim	BIO8	Bioclimatic	Mean temperature of wettest quarter
WorldClim	BIO9	Bioclimatic	Mean temperature of driest quarter
WorldClim	BIO12	Bioclimatic	Annual precipitation
WorldClim	BIO15	Bioclimatic	Precipitation seasonality
SSURGO	erodibility	Geological	Susceptibility of soils to erosion
SSURGO	albedo	Geological	Reflective property of surface
LANDFIRE	LC20_CC_200	Biotic	Forest canopy cover
LANDFIRE	LC20_CBH_200	Biotic	Forest canopy base height
LANDFIRE	LC16_EVH_200	Biotic	Existing vegetation height
LANDFIRE	LC16_EVC_200	Biotic	Existing vegetation cover
The National Map	Elevation	Geographical	Elevation from sea level
Calculated from Elevation Layer	Slope	Geographical	Slope of surface
Calculated from Elevation Layer	Aspect	Geographical	Direction land faces

Environmental niche models, sometimes referred to as species distribution models, of *P.c.clarkii* and *P.c.nantahala* were generated with the R package ENMTools 1.0 (Warren et al. 2021). For each taxon, niche models were generated with all three datasets using Maxent and the generalized linear model (GLM) method in ENMTools. Relative contribution of variables to each model was determined by model-specific variable importance analysis with the command “enmtools.vip” and the “permute” method in ENMTools. Niche models and variable importance plots were plotted in R. We used the “identity.test” function of ENMTools to test whether the niche of each putative subspecies was significantly different. Tests were done with 100 replicates and 10,000 background points. The niche overlap metrics D (Schoener 1968) and I (Warren et al. 2018) were used in significance tests with a critical value of 0.05.

### ﻿Data and code availability

All scripts are available from https://github.com/nathanwhelan/Patera. STACKS output, alignments, COI distance matrix, tree files, SNAQ input and output, shell photographs, and environmental data raster files are available on FigShare https://doi.org/10.6084/m9.figshare.19638642. Demultiplexed and decloned 3RAD data are available from NCBI SRA BioProject PRJNA944142.

## ﻿Results

### ﻿Molecular analyses

Sanger sequencing for all three genes was successful for three *P.perigrapta* individuals, six *P.c.clarkii*, three *P.c.nantahala*, and one potential hybrid individual with an intermediate morphology (i.e., P.aff.clarkii 008; Fig. 4D; Table 1). We were able to successfully sequence nuclear genes, but not COI, for one additional *P.c.clarkii* individual. For 3RAD sequencing, after demultiplexing and clone filtering, the number of raw paired-end reads per individual ranged from 256,990 to 1,899,741 (average = 1,018,463). After filtering, 2,905 loci were retained. Loci had an average length of 273 bp, and 74.8% of loci had overlapping read pairs. The number of SNPs per locus ranged from 1–41, with an average of 14 SNPs per locus.

**Figure 6.**
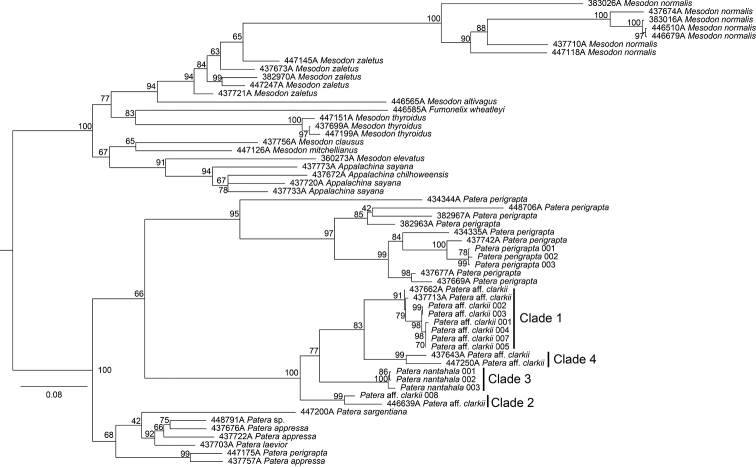
COI maximum likelihood tree. Branches are labelled with ultrafast bootstrap support. Clades within *P.clarkii* s.l. are labelled with numbers referred to in the main text. Scale is in substitutions per site.

The COI tree had greater taxon sampling than other analyses because only COI data were available for *Patera* and related Polygyridae from previous studies. Generally, deep divergences were inferred within putative species and multiple species were not monophyletic (Fig. 6). This could be the result of misidentifications, taxonomy in need of revision, or more likely both. One individual of “*P.perigrapta*”, (447175A) that was sequenced by Perez et al. (2014), was placed sister to an individual of *Pateraappressa* (Say, 1821), indicating that 447175A was most likely misidentified.

*Pateraclarkii* s.l. was recovered in four main clades on the COI tree, all of which had ultrafast bootstrap support greater than 90 (see labels on Fig. 6). All sequenced individuals that we initially identified as *P.c.clarkii* (i.e., excluding the possible hybrid), were in a clade with two individuals sequenced by Perez et al. (2014) that were collected from northern Georgia (Clade 1, Fig. 6). This clade was sister to two additional *P.c.clarkii* individuals from Perez et al. (2014) that were also collected in northern Georgia (Clade 4, Fig. 6). Clade 3 contained three *P.c.nantahala* individuals and was sister to Clades 1 and 4 (Fig. 6). The sister clade to all other *P.clarkii* s.l. contained the individual that we initially hypothesized to be a hybrid (individual P.aff.clarkii 008) and an individual from eastern Tennessee that was identified as *P.clarkii* by Perez et al. (2014; Clade 2, Fig. 6). The “aff.” epithet is used hereafter because both “*P.c.clarkii*” lineages resemble the type, but we are unable to determine which lineage, if either, is true *P.c.clarkii* (see below). Relationships among the four clades within *P.clarkii* s.l. had limited support on the COI gene tree. However, pairwise distances among *P.clarkii* s.l. clades were high, ranging from 9.3%–12.5%. Automatic species delimitation analysis with ASAP indicated the presence of four putative species, corresponding to the four main clades of *P.clarkii* s.l. on the COI tree (Fig. 6).

**Figure 7.**
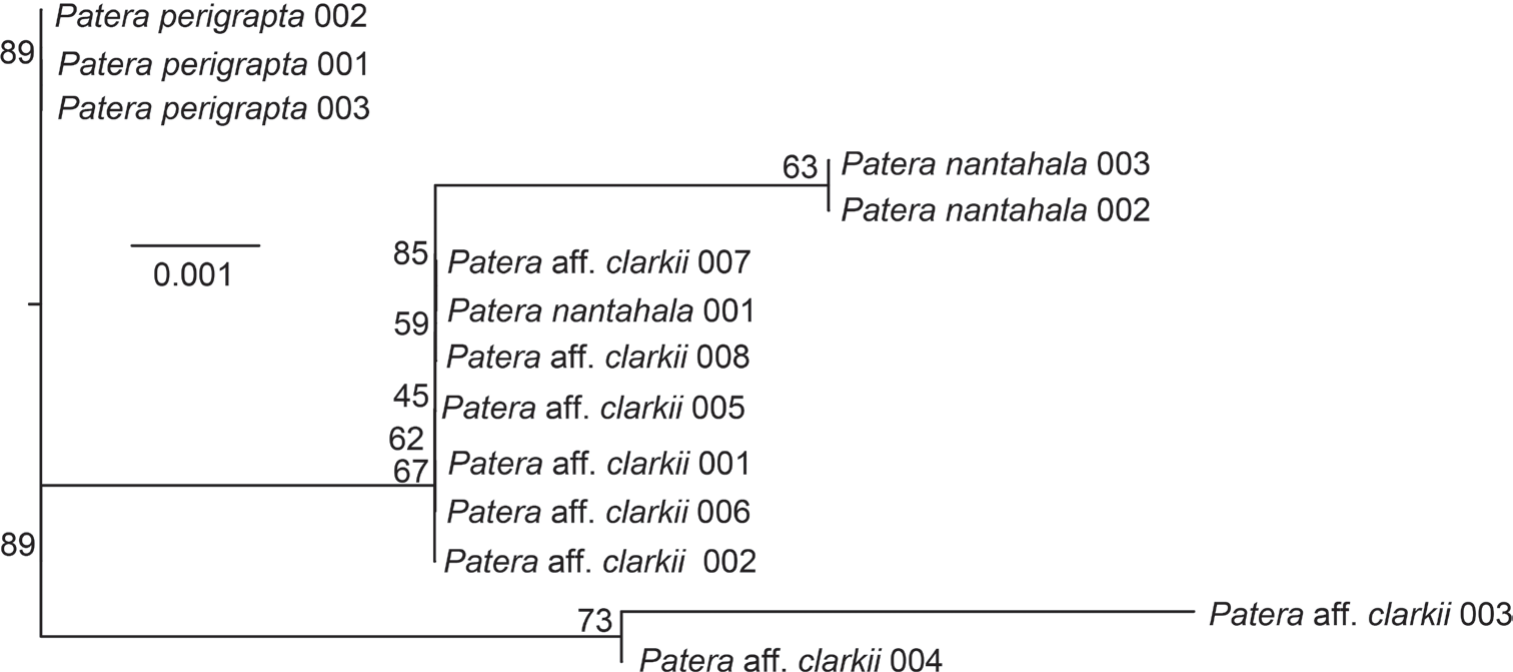
H3 maximum likelihood tree. Branches are labelled with ultrafast bootstrap support. Scale is in substitutions per site.

In contrast to the COI tree, there was virtually no resolution on the H3 gene tree as no node had greater than 89% ultrafast bootstrap support (Fig. 7). Two *P.c.nantahala* individuals had a private H3 allele and were sister to each other on a long branch within a clade consisting of most *P.clarkii* s.l. individuals. Two *P.c.clarkii* individuals were sister to all other *P.clarkii* s.l. All individuals that we sequenced had the same 28S sequence, including the three *P.perigrapta* individuals that served as outgroups.

**Figure 8.**
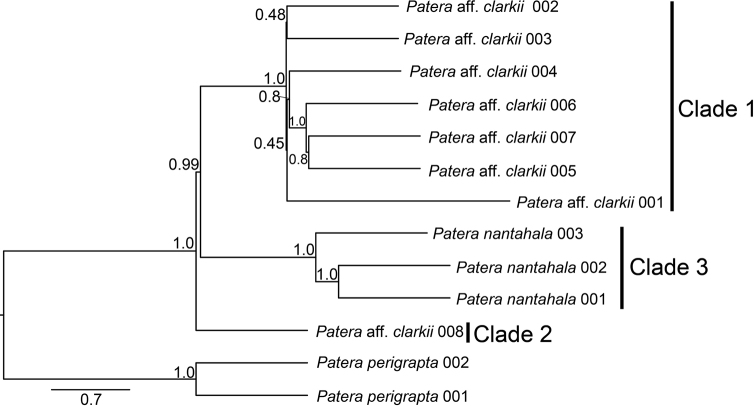
ASTRAL species tree. Branches are labelled with local posterior probability. Clades are labelled with numbers referred to in the main text. Scale is in coalescent units.

Three *P.clarkii* s.l. clades were resolved on the ASTRAL species tree, each having 100% local posterior probability (Fig. 8). The absence of a clade on the ASTRAL tree corresponding to Clade 4 on the COI tree was likely a result of the two Clade-4 individuals from Perez at al. (2014) not being available for genome-based ASTRAL analysis. ASTRAL clades were given designations 1, 2, and 3 to match those on the COI tree and distinguish between the two *P.aff.c.clarkii* lineages and *P.c.nantahala*. The clade with the individual initially identified as a possible hybrid between *P.c.clarkii* and *P.c.nantahala* (i.e., individual P.aff.clarkii 008) was placed in “Clade 2”, whereas other *P.c.clarkii* were placed in “Clade 1”. Relationships among clades on the ASTRAL species tree were congruent with the COI mitochondrial gene tree (Figs 5, 8), albeit without individuals from Perez et al. (2014) on the ASTRAL tree. Analyses with SNAQ indicated that the data were tree-like as the zero-reticulation model and one-reticulation model had similar pseudo log-likelihood values (Suppl. material 2: fig. S1). Expected versus observed concordance factor plots were also similar between models with no obvious outliers on the zero-reticulation model compared to the one-reticulation model (Suppl. material 2: fig. S2). The reticulation event on the one-reticulation network had a gamma value of less than 3.5 (Suppl. material 2: fig. S3). Thus, SNAQ analyses rejected our hypothesis that individual P.aff.clarkii 008 was a hybrid. SNAQ analyses also rejected recent or ongoing gene flow among *P.clarkii* s.l. clades, including among *P.c.nantahala* and the *P.aff.c.clarkii* clades.

### ﻿Shell shape and size variation

The final morphometric dataset had 21 individuals of *P.c.nantahala* and 77 *P.c.clarkii* (Suppl. material 1). We were unable to separate the *P.aff.c.clarkii* lineages for geometric morphometrics because we only had sequence data for one individual from Clade 2 and geographic and morphological characters to distinguish the *P.aff.c.clarkii* lineages have not been documented and may not exist. This likely resulted in a greater breadth of shape for *P.c.clarkii*, thereby increasing the chance of shape overlap between *P.c.clarkii* and *P.c.nantahala*.

Geometric morphometrics confirmed what was mostly evident by eye. Procrustes ANOVA indicated a significant size and shape difference between *P.c.nantahala* and *P.c.clarkii* (*p* < 0.0001; Table 3). Canonical variate analysis indicated that 100% of shell shape variation was explained by a single axis and there was no overlap between the two subspecies (Fig. 5; Table 3). Permutation tests indicated significant differences in shell shape between *P.c.clarkii* and *P.c.nantahala* (p < 0.0001). *Paterac.nantahala* had a wider shell and a more compressed spire height compared to overall width (Fig. 5). The parietal tooth in *P.c.nantahala* did not protrude as far as in *P.c.clarkii* (Fig. 5).

**Table 3.** Results of geometric morphometric statistical tests.

**Procrustes ANOVA for Shape**							
Effect	Procrustes Sum of Squares	Procrustes mean squares	degrees of freedom	Goodall’s *F*	*p* (F)	Pillai’s trace	*p* (Pillai’s trace)
Species	0.080648	0.004032	20	11.98	< 0.0001	0.76	< 0.0001
Residual	0.646333	0.000337	1920				
**Procrustes ANOVA for Centroid Size**							
Effect	Procrustes Sum of Squares	Procrustes mean squares	degrees of freedom	Goodall’s *F*	*p* (F)		
Species	6.258875	6.258875	1	73.29	< 0.0001		
Residual	8.19893	0.085398	96				
**Canonical Variate Analysis**							
Eigenvalues	% Variance	Mahalanobis distance between species	*p* (Mahalanobis distance)	Procrustes distance between species	*p* (Procrustes distance)		
3.180948	100	4.302	<0.0001	0.0699	< 0.0001		

Several qualitative morphological differences distinguish *P.c.nantahala* from *P.c.clarkii*. The denticle on the baso-palatal wall is much more prominent in *P.aff.c.clarkii* Clade 1 individuals (Fig. 3) and slightly more prominent in the *P.aff.c.clarkii* individual from Clade 2 (Fig. 4D) than in *P.c.nantahala* (Figs 2, 4A–C). Furthermore, the parietal tooth covers a larger part of the aperture in both P.aff.clarkii Clades 1 and 2 (Figs 3, 4D) compared to *P.c.nantahala* (Figs 2A, 4A–C). In two of the three sampled *P.c.nantahala* individuals, mantle pigmentation displayed a branching pattern (Fig. 4A, B), versus a horizontal band in *P.c.clarkii* clades when present (Figs 3A–D, F, 4D). However, the branching pattern does not appear to be diagnostic as it is not visible on the types (Fig. 2), nor on one individual we collected (Fig. 4C).

### ﻿Environmental niche models and niche overlap

After removal of several suspect records, occurrence data consisted of nine records for *P.c.nantahala* and 79 for *P.c.clarkii*. Spatial rarification of the data resulted in a reduced dataset with three records for *P.c.nantahala* records and 46 for *P.c.clarkii* (Suppl. material 1).

**Figure 9.**
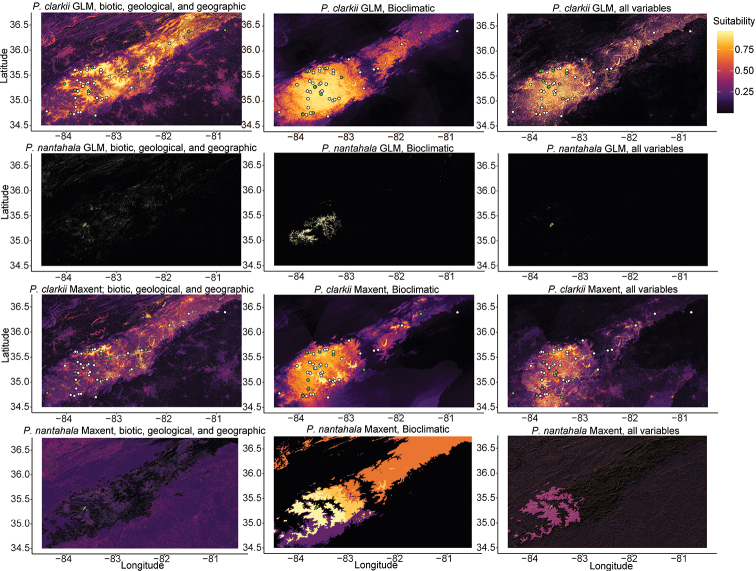
Environmental niche models for *P.clarkii* and *P.nantahala*. Brighter colors indicate locations with greater niche suitability.

Environmental niche models inferred with Maxent resulted in much greater predicted suitable habitat for *P.c.nantahala* compared to GLMs, whereas models for *P.c.clarkii* were similar regardless of modeling method (Fig. 9). The relative importance of any given variable was highly dependent on the modeling approach (i.e., Maxent vs. GLM), the variables included, and whether *P.c.nantahala* or *P.c.clarkii* was being modeled (Suppl. material 2: figs S4–S15). Models inferred with only BioClim variables resulted in considerably greater predicted suitable habitat for *P.c.nantahala* than models that used all environmental variables. Similarly, models that used all variables appeared to be least likely to overpredict suitable habitat of *P.c.clarkii* based on its known range (Fig. 9). *Paterac.nantahala* only occupied locations that were classified on the SSURGO soil map units as “Inceptisols: Sylco-Cataska complex, 50 to 95 percent slopes, very rocky”. Inceptisols are characterized as being from humid and subhumid regions with subsurface soil layers lacking illuviated material and without an ochric epipedon (Soil Survey Staff 1999). Some records of *P.c.clarkii* were also from locations classified as “Inceptisols: Sylco-Cataska complex, 50 to 95 percent slopes, very rocky”. However, these records were separated from *P.c.nantahala* by at least one different soil type, and *P.c.clarkii* also occupied other soil types, including ultisols (i.e., soils with low base saturation and kandic or argillic horizons, generally with a vegetation of coniferous or hardwood forests) and entisols (i.e., soils with little or no evidence of layers). For more details on soil types see Soil Survey Staff (1999).

Overlap comparisons indicated significant differences in niches of *P.c.clarkii* and *P.c.nantahala* when using GLMs and datasets with non-bioclimatic variables (*p* < 0.05; Table 4). Although not statistically significant, D and I values were also low for GLMs with only bioclimatic variables (Table 4). Maxent models did not indicate significant differences (*p* > 0.05), except with the D statistic on the model that was generated with all environmental variables (Table 4). However, the GLM with only bioclimatic variables and all Maxent models clearly overpredicted suitable habitat for *P.c.nantahala* (Fig. 6), which is why we emphasize the significant results. The *P.c.clarkii* records included in environmental niche modeling potentially include more than one species (i.e., the multiple molecular P.c.aff.clarkii lineages from Clade 1, Clade 2, and Clade 4; Figs 3, 4D, 6), but inferred niche differences should be robust as inclusion of the multiple lineages from GBIF records of “*P.c.clarkii*” is likely to increase and homogenize predicted habitat of *P.c.clarkii*, which would have resulted in overestimating niche overlap.

**Table 4.** D and I environmental niche overlap metrics for GLM and Maxent based niche overlap tests. Bold values indicate models with significant niche differences between *P.clarkii* and *P.nantahala* at α = 0.05.

	Non-bioclimatic variables	Bioclimatic variables	All environmental variables
GLM: D	**0.013**	0.033	**0.001**
GLM: I	**0.089**	0.166	**0.026**
Maxent: D	0.334	0.361	**0.163**
Maxent: I	0.627	0.659	0.371

### ﻿Systematics

**Family Polygyridae Pilsbry, 1895**

**Subfamily Triodopsinae Pilsbry, 1940**

**Tribe Mesodontini Tryon, 1866**

#### ﻿Genus Patera

Taxon classificationAnimaliaStylommatophoraPolygyridae

Albers, 1850

996FCAE7-CA1F-594C-941A-B7E47191C05F

Helix (Patera) Albers, 1850: 96. Type species: Helixappressa Say, 1821, by subsequent designation (Pilsbry 1930: 326) [non Patera Lesson, 1839 (Cnidaria)].

##### Remarks.

*Patera* is a junior homonym of *Patera* Lesson, 1839 (Cnidaria). However, *Patera* Lesson, 1839 has only been used in a few treatises during the 19^th^ century and at the beginning of the 20^th^ century, whereas *Patera* Albers, 1850 is in widespread use. As such, continued usage of the junior homonym is in the best interest of stability and the case should be referred to the International Commission on Zoological Nomenclature for a ruling under Art. 23.9.3 of the Code (ICZN 1999).

#### ﻿ Patera nantahala

Taxon classificationAnimaliaStylommatophoraPolygyridae

(Clench & Banks, 1932)

992D61A6-3561-5E91-BDC1-8563BF1DC171

Polygyra (Triodopsis) nantahala Clench & Banks, 1932: 17, pl. 2, figs 1–3, 5.
Mesodon clarki nantahala –Pilsbry 1940: 731, fig. 440g; Chambers 1981: 55–59; Hubricht 1983: 13; Hubricht 1985: 44; Richardson 1986: 45.
Patera clarki nantahalae [sic]–Emberton 1995: 72.

##### Type material.

***Holotype***: MCZ 86429. GS Banks leg., 27 August 1930.

***Paratypes***: ANSP 153664 (4 spms), GS Banks leg., 25 August 1930; MCZ 82533 (1 spm), Clench, Archer & Rehder leg., 7 August 1931; MCZ 185877 (3 spms), Clench, Rehder & Archer leg., July 1931, ex. A Archer collection; USNM 408310 (3 spms), Clench, Rehder & Archer leg., 1931.

##### Type locality.

Blowing Springs, cliff ridges, Nantahala Gorge, Swain County, North Carolina.

##### Other material examined.

**USNM 1522409, USNM 1522410**: Adjacent to unnamed tributary of Nantahala River, southeast cliff of Nantahala Gorge, Swain County, North Carolina, 35.308, -83.644, GenBank: OQ617122, OQ628062, OQ628460, OQ617123, OQ628056, OQ628461, SRA: SRX19664333, SRX19664332; **USNM 1522411**: Adjacent to Pizza by the River, northeast corner of Nantahala Gorge, Swain County, North Carolina, 35.336, -83.620, GenBank: OQ617124, OQ628055, OQ628462, SRA: SRX19664329 **ANSP 171736**: Blowing Springs, Swain County, North Carolina; **ANSP 348077**: Nantahala Gorge, Swain County, North Carolina, 35.40, -83.25; **MCZ 94130**: Blowing Springs, Nantahala Gorge, Swain County, North Carolina.

##### Diagnosis.

Shell imperforate, subglobose, weakly translucent, with 5.5–5.75 whorls. Teleoconch sculpture of coarse, prosocline, axial striae. Spire low, dome-shaped, sutures weakly impressed. Aperture lunate, peristome white, with small basal notch. Slightly curved parietal tooth, moderate in size for the genus. Mantle pigmentation of branching lines in at least some individuals.

##### Distribution.

Restricted to the eastern slope of the Nantahala Gorge in North Carolina, USA.

##### Ecology.

Little is known about the ecology of *P.nantahala*. The species appears to prefer the moist, highly vegetated habitats that receive little sunlight, which are typical of the eastern slope of the Nantahala Gorge. Found only in habitats with soil characterized by the SSURGO soil map as “Inceptisols: Sylco-Cataska complex, 50 to 95 percent slopes, very rocky”.

##### Conservation status.

Federally threatened under the U.S. Endangered Species Act. Listed as threatened by the state of North Carolina. Available data indicate that *P.nantahala* is in one of the three “threatened” IUCN ranking categories, likely falling under “endangered”.

##### Remarks.

All sampled *P.nantahala* individuals are more similar to the holotype and paratypes of *P.nantahala* than to the types of *P.clarkii*. Shell shape of *P.nantahala* differs significantly from closely related lineages (Figs 2–5; Table 3). We are unable to comment on internal anatomical variation among P.aff.clarkii lineages and *P.nantahala* because we did not preserve specimens in a manner suitable for anatomical work. Emberton (1995) examined internal anatomy of *Patera* and found no differences among *P.clarkii*, *P.perigrapta*, and other species in the subgenus Patera (Patera), making it unlikely that anatomical investigations would yield diagnosable features among the lineages examined here.

## ﻿Discussion

Our results demonstrate that *P.nantahala* is a distinct species based on molecular, morphological, and ecological data. Recognition of *P.nantahala* renders *P.clarkii* polyphyletic, and our phylogenetic analyses indicate that unrecognized species diversity still exists within *P.clarkii* s.l. Recognition of *P.nantahala* at the rank of species is also consistent with the framework developed by Horsáková et al. (2019) for recognizing “cryptic” species in terrestrial snails, who argued that multiple lines of evidence including mitochondrial and nuclear concordance, quantitative morphological differences, and ecology should support a taxonomic hypothesis before recognizing entities at the species level. In contrast, a better understanding of the geographic ranges of the P.aff.clarkii lineages and establishing which lineage should be ascribed to *P.clarkii* s.s. is needed before a new species can be described. Our results emphasize the need for genome-based analyses to understand diversity and conservation of North American terrestrial snails. From a conservation standpoint, the original listing decision under the Endangered Species Act treated *P.nantahala* as a distinct entity. Thus, our results support continued protection.

### ﻿Species, morphological, and genetic diversity

Both mitochondrial and 3RAD data are congruent and demonstrate that *P.nantahala* is reciprocally monophyletic with respect to P.aff.clarkii lineages. Mitochondrial divergence among *P.nantahala* and P.aff.clarkii lineages exceeds 9%, and both SNAQ and mitochondrial analyses indicate a lack of recent nuclear introgression. Thus, *P.nantahala* is a distinct evolutionary lineage.

The observed absence of recent gene flow is unlikely to be a result of sampling error as sampling locations for *P.clarkii* and *P.nantahala* were in close proximity and within likely contact zones. Furthermore, if gene flow was currently occurring, we would not expect divergence patterns on the mitochondrial tree and ASTRAL species tree to be congruent and to match morphological differences. Although some may argue that additional sampling of *P.nantahala* would be desirable prior to revising its status, this is not preferable given its conservation status. Destructive sampling of museum specimens is not a suitable alternative given the paucity of preserved specimens and because techniques for 3RAD with dry shell material are unproven. Furthermore, network-based approaches with genomic data are sufficiently sensitive to assess gene flow, even with one or two individuals per species (Solís-Lemus and Ané 2016; Mao et al. 2018; Watson et al. 2020).

The branching pattern inferred in phylogenetic analyses supports the presence of several unrecognized species. Analysis with ASAP indicated that Clades 1–4 on the COI tree were each a distinct species (Fig. 6). As noted above, ASAP is not based on the multispecies coalescent, but rather barcode gaps, which has been shown to be more conservative in splitting entities into hypothesized species than other automatic delimitation methods (Strong and Whelan 2019). Nevertheless, automatic species delimitation methods can give incongruent results, and the best automatic approach for land snails, if there is one, is unclear (Sauer and Hausdorf 2011; Greve et al. 2012; Prévot et al. 2013; Bamberger et al. 2022).

The absence of gene flow among *Pateraclarkii* s.l. lineages inferred with SNAQ also corroborates ASAP results. Notably, SNAQ found no gene flow between individual “P.aff.clarkii 008” (i.e., Clade 2; Figs 6, 8) with other clades, therefore rejecting our initial hypothesis that individual “P.aff.clarkii 008” was a hybrid between *P.clarkii* and *P.nantahala*. However, we refrain from describing a new species pending additional work to determine its geographic range. Furthermore, we are unsure whether Clade 1, 2, 4, or an unsampled lineage, represents true *P.clarkii* because phylogenetic analyses did not include individuals from the type locality Tuskee [sic, Tuskeegee] Cove, Cherokee County [now Graham County], North Carolina. However, individuals sampled closest to the type locality were in Clade 1. Topotypic material of the other available species-group name currently in the synonymy of *P.clarkii* is also needed (i.e., Polygyraclarkiivar.bradleyi Vanatta 1912) prior to species descriptions. We note that *P.clarkii* is the correct original spelling and should be preserved under Article 31.1.3 of the Code (ICZN, 1999) even though “*P. clarki*” is more commonly used in the recent literature.

Geometric morphometrics showed that *P.nantahala* has a significantly different shell shape compared to closely related congeners. We were unable to unambiguously assign museum records to one of the three Pateraaff.clarkii lineages because distinguishing shell features or geographic ranges have not been established. Future studies with more *P.clarkii* s.l. sampling for molecular phylogenetics will be necessary to allow confident clade assignments that can be used in geometric morphometrics. However, truly cryptic species may exist within *Patera*.

Hubricht (1983) claimed that *P.clarkii* exists in the Nantahala Gorge and *P.nantahala* exists outside the Nantahala Gorge. These conclusions were based on comparisons of shell morphology, but the exact shell features, aside from shell size, used to support these conclusions were not reported. Phylogenetic analyses, geometric morphometrics, and environmental modeling results reject Hubricht’s (1983, 1985) hypothesis that *P.nantahala* is not a valid subspecies. Although we cannot completely rule out that future survey work will find overlap in the range of *P.clarkii* and *P.nantahala*, the absence of gene flow and high genetic divergence indicate that the two species are reproductively isolated.

Our results add to a growing body of research that used genomic tools to better understand terrestrial snail evolution (e.g., Razkin et al. 2016; Phillips et al. 2020; Bober et al. 2021; Bamberger et al. 2022). When used in conjunction with distributional, ecological, and morphological data, as done here, genomic data appear especially well-suited for resolving polygyrid relationships. Our conclusions about species diversity likely would have been different, and incorrect, if we had relied only on 28S and H3 for nuclear genetic data. For instance, the H3 tree indicated little genetic differentiation among *P.clarkii* s.l. lineages (Fig. 7), and 28S was invariant across *P.perigrapta*, *P.nantahala* and *P.clarkii* s.l. Prior to generating nuclear data via 3RAD sequencing, we thought gene flow among sampled *P.clarkii* s.l. was possible, if not probable, based on the 28S and H3 data. In contrast, 3RAD data indicate that incomplete lineage sorting, rather than gene flow, is responsible for a lack of resolution in the 28S and H3 genes. This finding is essential for future research on polygyrids, and we encourage future studies to employ genomic data for population- and species-level research.

### ﻿Environmental niche models

The environmental niches of *P.nantahala* and *P.clarkii* are significantly different according to GLM analyses with non-bioclimatic data included, which appear to be the most accurate given environmental niche model plots and known ranges (Fig. 9). For example, predicted suitable habitat using GLMs appears reasonable and not overpredicted, particularly when all environmental variables were used. We hypothesize that niche models with non-bioclimatic variables are more accurate because of unique abiotic features of the southeastern slope of the Nantahala Gorge. Maxent models for *P.nantahala* predicted suitable habitat far outside the known species range and in locations where only shells that match the morphology of *P.clarkii* have been recorded. Thus, disagreement in the significance of environmental niche differences between GLM and Maxent analyses appears to be a result of Maxent making overpredictions in the suitable habitat of *P.nantahala* (Fig. 9).

Our results indicate the need to be cautious when using environmental niche modeling approaches for understudied, narrow-range endemics. Most analyses overestimated the distribution of *P.nantahala* (Fig. 9), and we do not think that suitable habitat inferred with Maxent represents true suitable habitat or an unrecognized, potential niche for *P.nantahala*. Furthermore, models that used only bioclimatic data performed worse, especially with *P.nantahala* (Fig. 9). We argue that overestimation of environmental niche is at least possible, if not likely, for any narrow range endemic, especially when relying entirely on bioclimatic data. Most environmental data used in niche modeling, particularly BioClim data, are likely not of adequate resolution for distinguishing the environmental niches of extreme narrow-range endemics. Our results indicate that if environmental niche models are to be generated for narrow-range endemics, environmental data other than bioclimatic variables are essential.

Environmental niche models that include data other than bioclimatic information can be useful for assessing the potential for narrow-range endemics to occupy other habitats, but they may not always be necessary to make inferences about terrestrial snail distributions and environmental niches. For example, even before running environmental niche models, SSURGO soil classifications of collection sites made clear that *P.nantahala* only inhabits a single, uncommon soil type, whereas *P.clarkii* s.l. inhabits many different soil types. More broadly, our results indicate that Maxent models will tend to overpredict ranges for narrow-range endemics. These findings should be applicable to other terrestrial snails.

## ﻿Conclusions

Morphological, ecological, and phylogenetic data support *Pateranantahala* as a valid species. We hypothesize that the ancestor of *P.nantahala* invaded the Nantahala Gorge, or became isolated in the gorge, and subsequently underwent allopatric speciation, with the Nantahala Gorge and Nantahala River serving as dispersal barriers. Although the recognition of *P.nantahala* is a step in the right direction, the systematics of Polygyridae requires comprehensive revision. Despite calls for increased study (Perez 2011; Perez et al. 2014), little progress has been made. Our results suggest that phylogenetically distinct lineages of polygyrids remain unrecognized. As such, species that may require conservation attention are being overlooked. This could lead to a loss of diversity and evolutionary potential before we know how many species of polygyrids exist. To improve polygyrid systematics, both increased sampling and genome-wide markers will be needed.

## Supplementary Material

XML Treatment for Patera

XML Treatment for Patera nantahala

## Supplementary materials

Supplementary material 1 Species information Data type table (excel document) https://binary.pensoft.net/file/840502This dataset is made available under the Open Database License (http://opendatacommons.org/licenses/odbl/1.0/). The Open Database License (ODbL) is a license agreement intended to allow users to freely share, modify, and use this Dataset while maintaining this same freedom for others, provided that the original source and author(s) are credited.Nathan V. Whelan, Ellen E. Strong, Nicholas S. Gladstone, Jason W. Mays

Supplementary material 2 Additional images Data type figures (PDF file) https://binary.pensoft.net/file/840503This dataset is made available under the Open Database License (http://opendatacommons.org/licenses/odbl/1.0/). The Open Database License (ODbL) is a license agreement intended to allow users to freely share, modify, and use this Dataset while maintaining this same freedom for others, provided that the original source and author(s) are credited.Nathan V. Whelan, Ellen E. Strong, Nicholas S. Gladstone, Jason W. Mays
