# Supplementary material for: ﻿Using genomics, morphometrics, and environmental niche modeling to test the validity of a narrow-range endemic snail, Pateranantahala (Gastropoda, Polygyridae)
Source: Zookeys. 2023 Apr 20;1158:91–120. doi: 10.3897/zookeys.1158.94152 (PMC10208601; doi:10.3897/zookeys.1158.94152)

Figure S1: Pseudo log-likelihood values of each network for models with different maximum reticulations (h). The drop between h = 0 and h = 1 is relatively large, but it represents a small change in pseudo log-likelihood value in absolute terms.

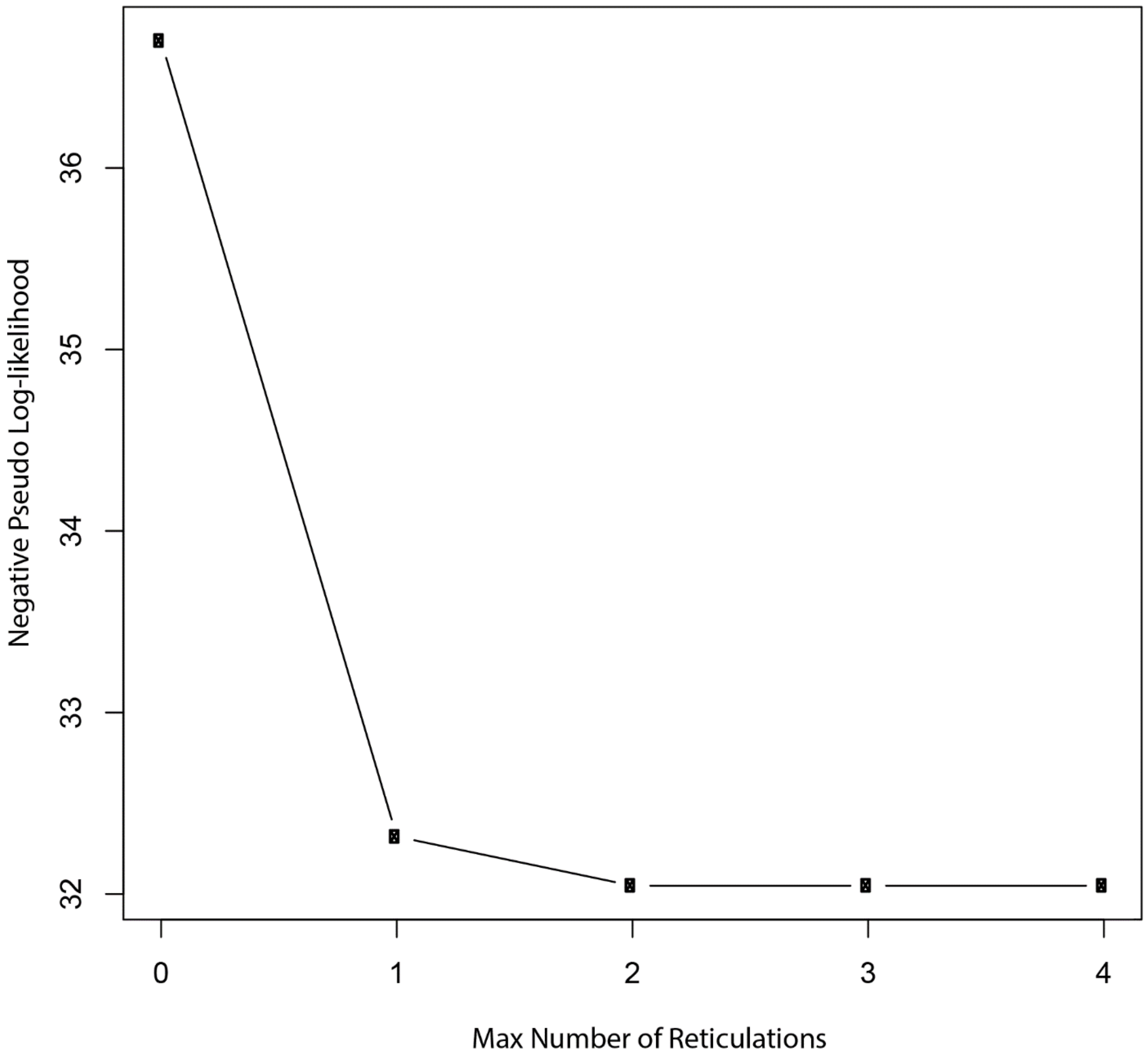

Figure S2: Observed versus expected CF

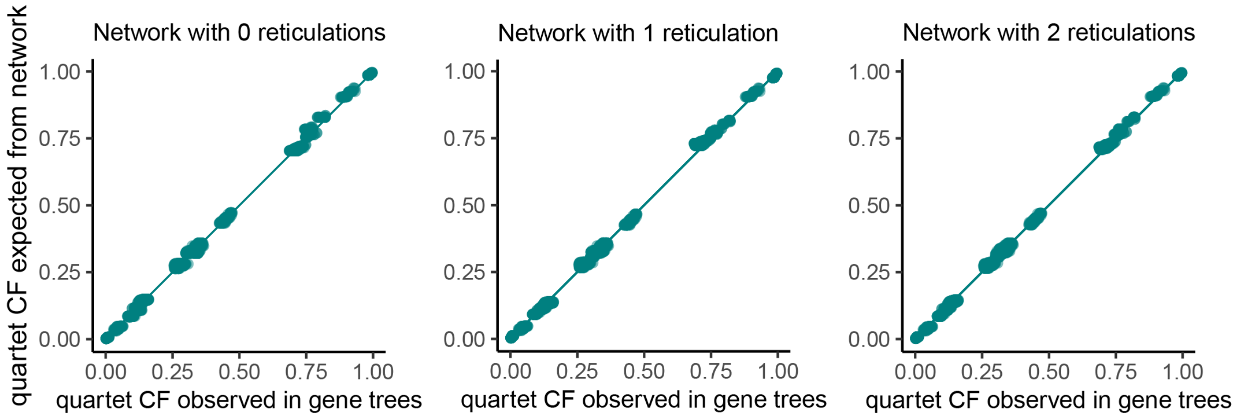

Figure S3: SNAQ network with one reticulation. Numbers along branches are gamma values for the reticulation event.

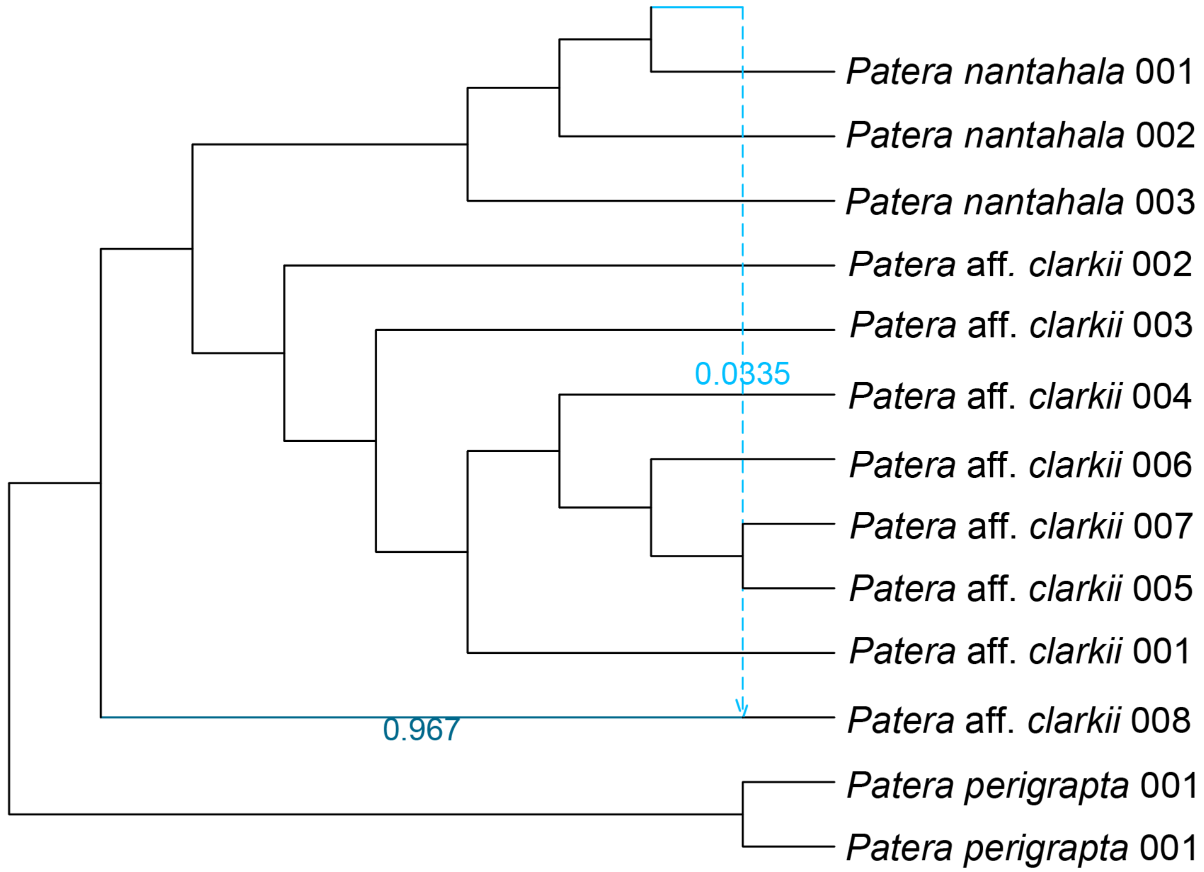

Figure S4: Variable importance plot for *P. clarkii* GLM niche model using biotic, geological, and geographic variables.

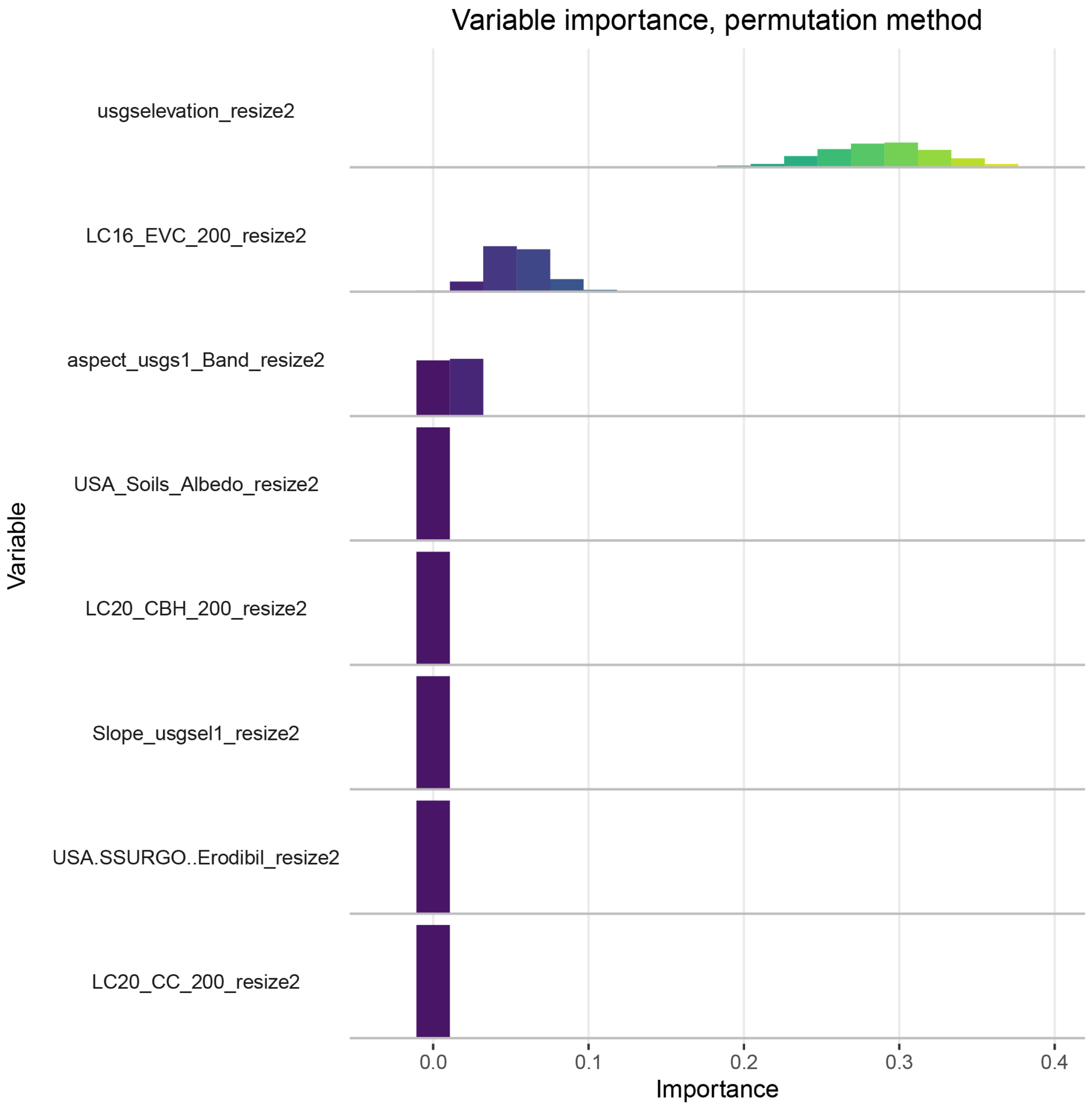

Figure S5: Variable importance plot for *P. clarkii* GLM niche model using only bioclimatic variables.

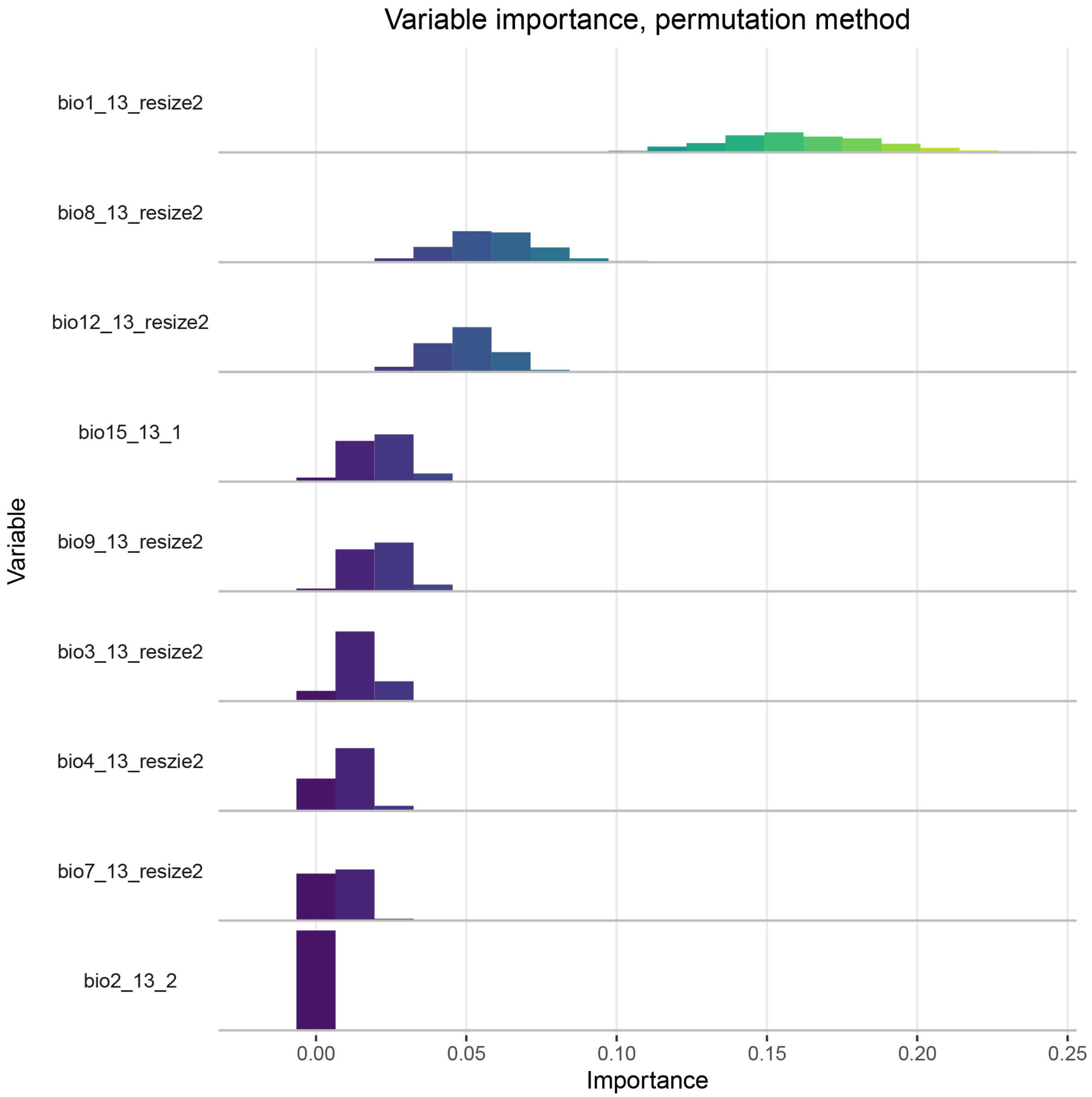

Figure S6: Variable importance plot for *P. clarkii* GLM niche model using all variables.

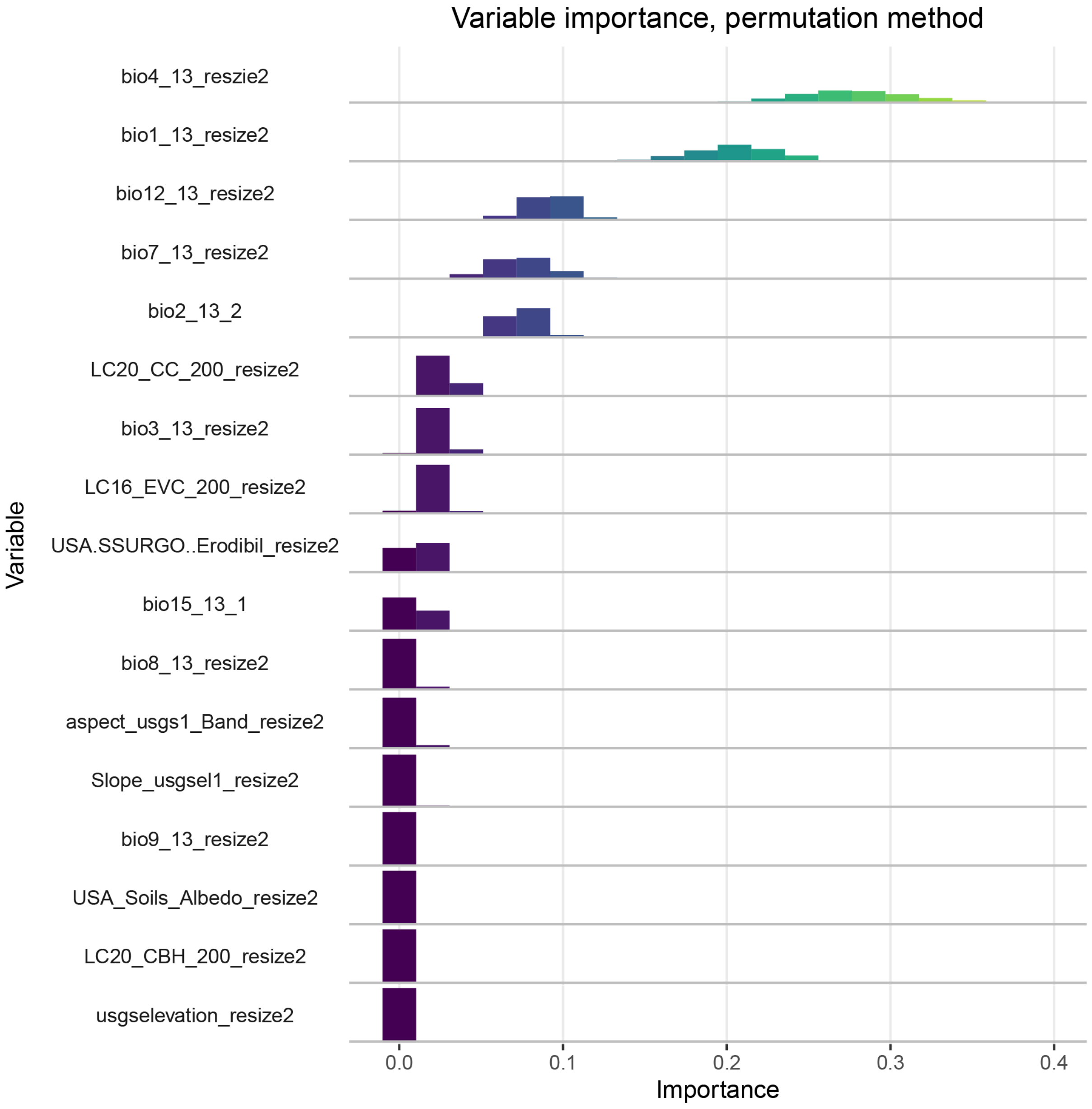

Figure S7: Variable importance plot for *P. nantahala* GLM niche model using biotic, geological, and geographic variables.

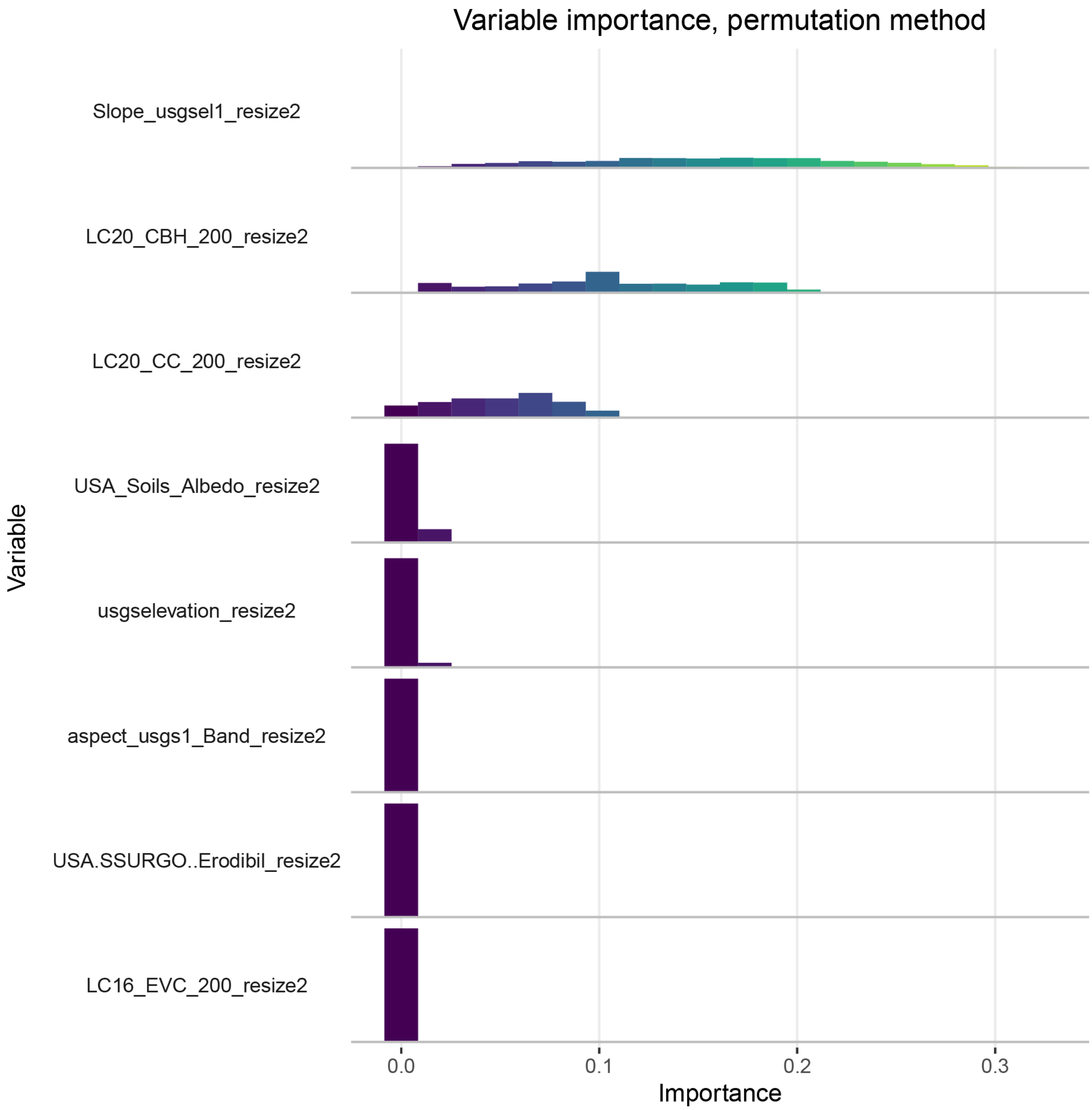

Figure S8: Variable importance plot for *P. nantahala* GLM niche model using only bioclimatic variables.

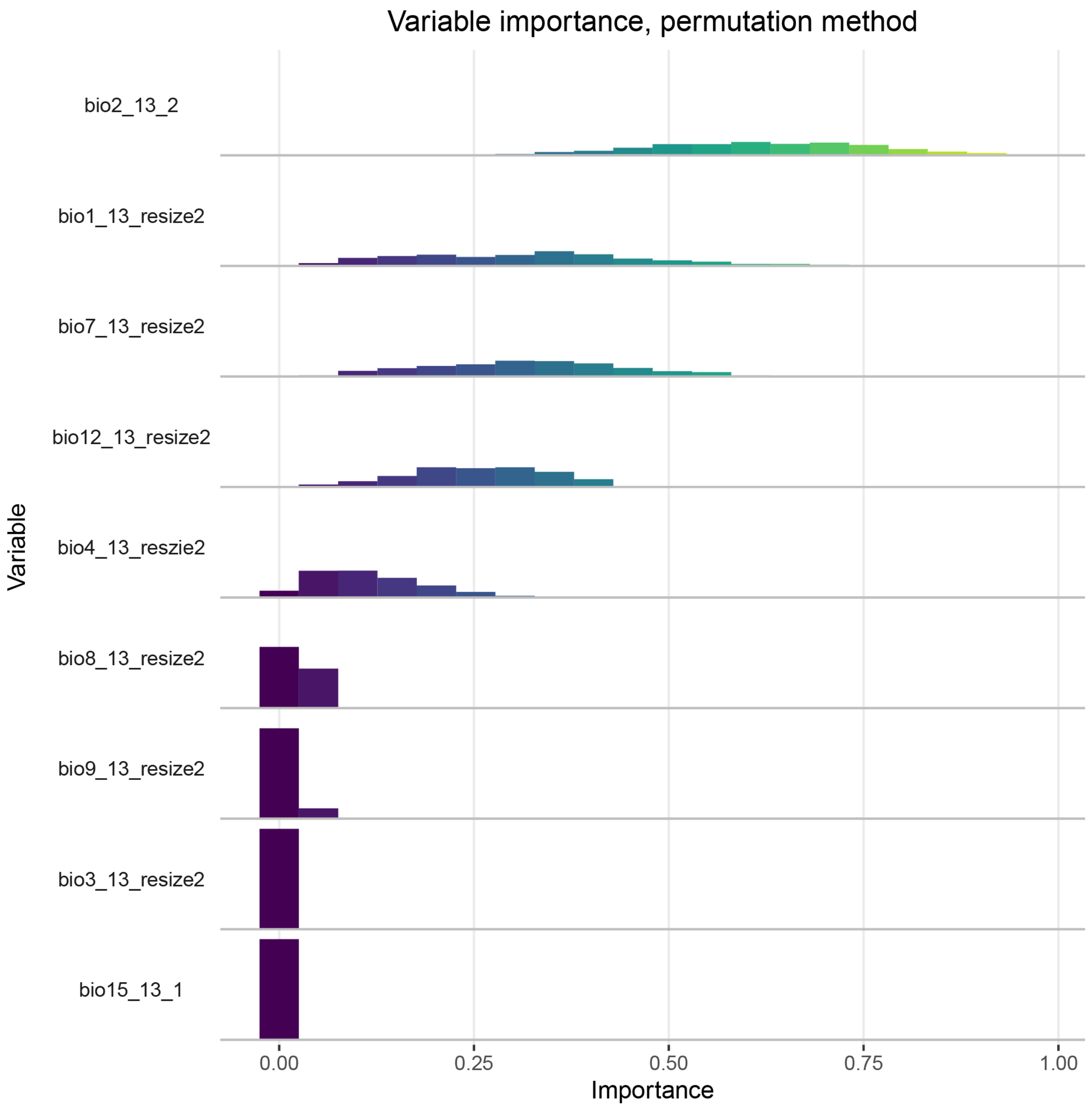

Figure S9: Variable importance plot for *P. nantahala* GLM niche model using all variables.

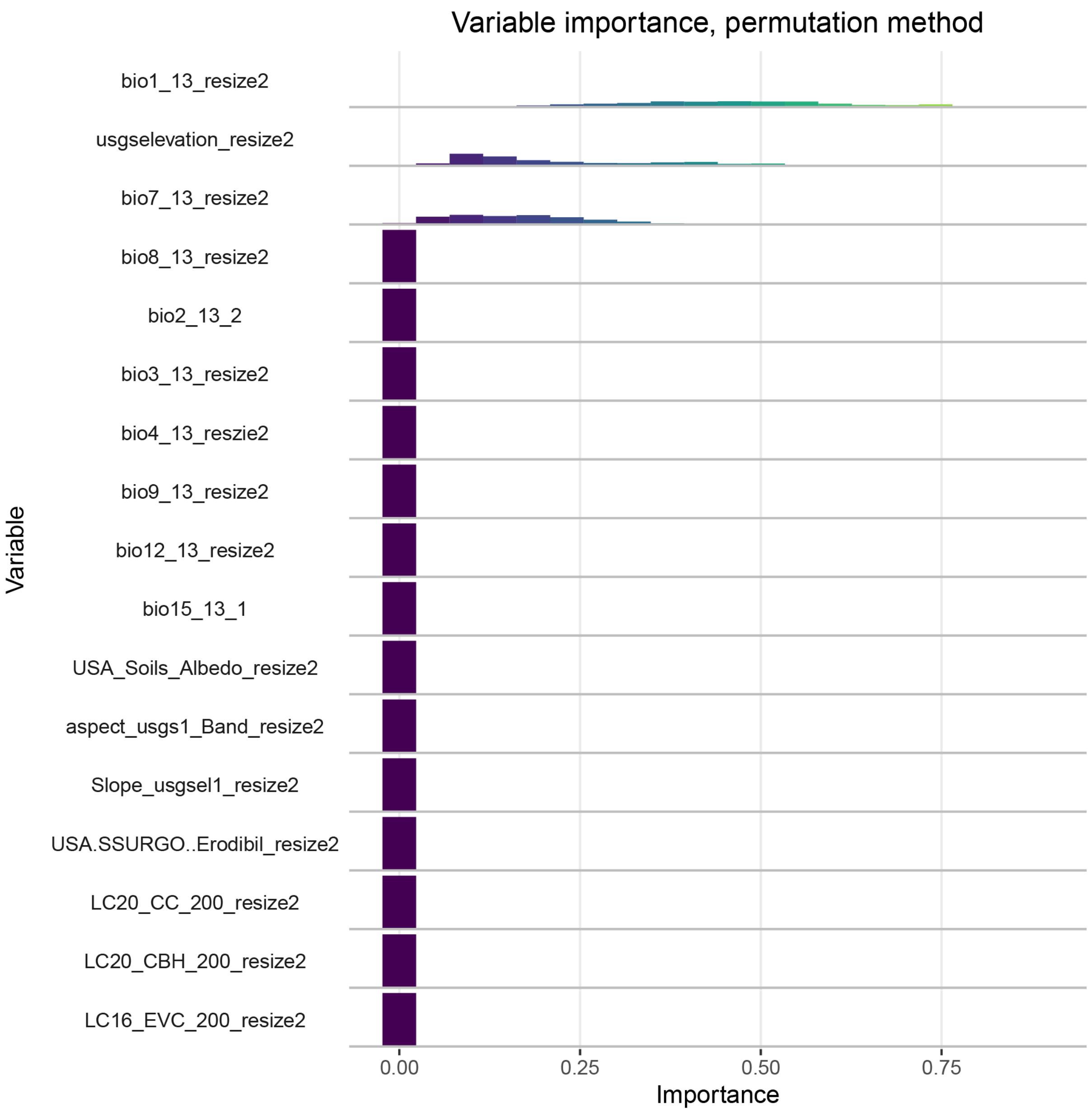

Figure S10: Variable importance plot for *P. clarkii* Maxent niche model using biotic, geological, and geographic variables.

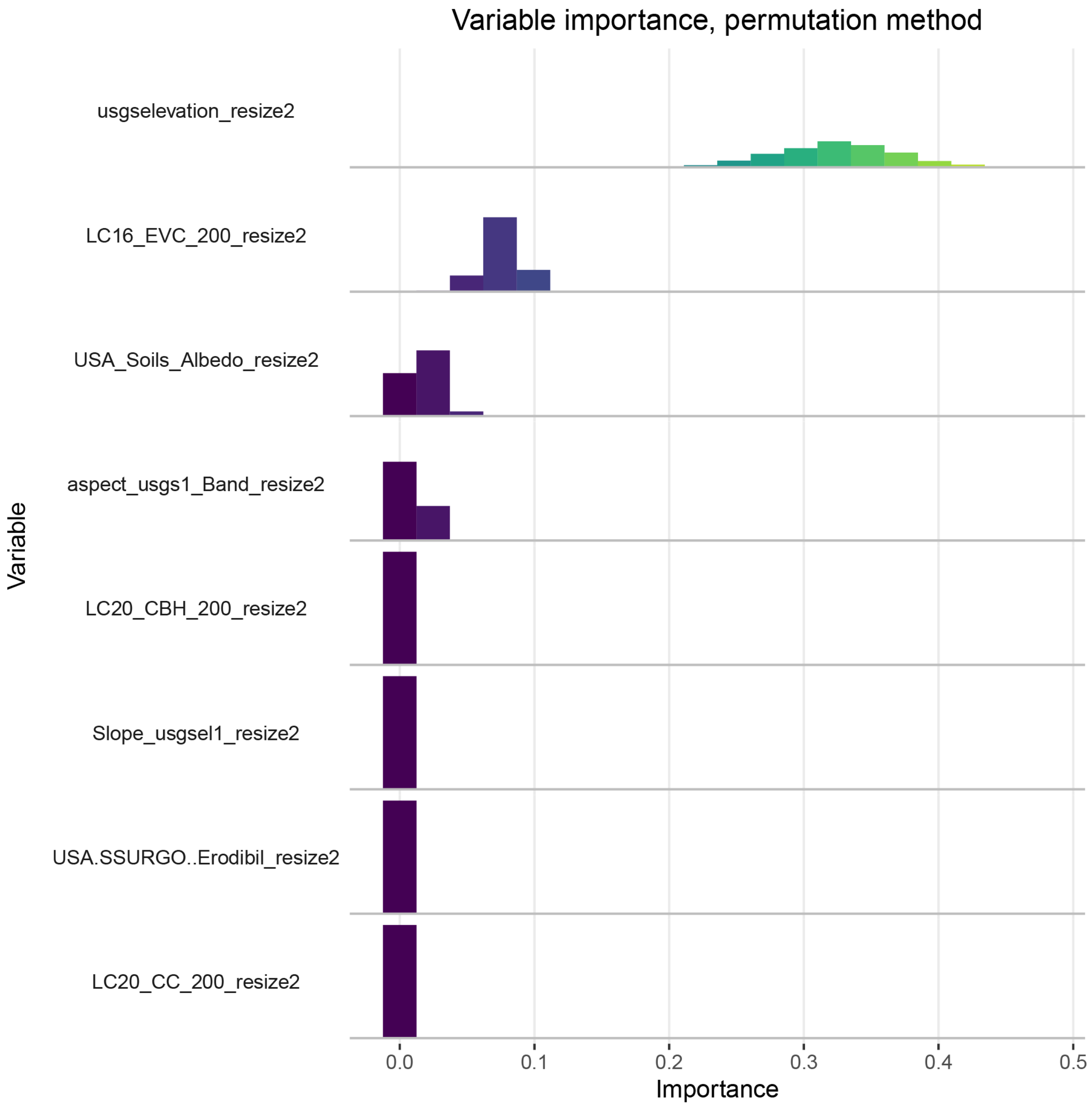

Figure S11: Variable importance plot for *P. clarkii* Maxent niche model using only bioclimatic variables.

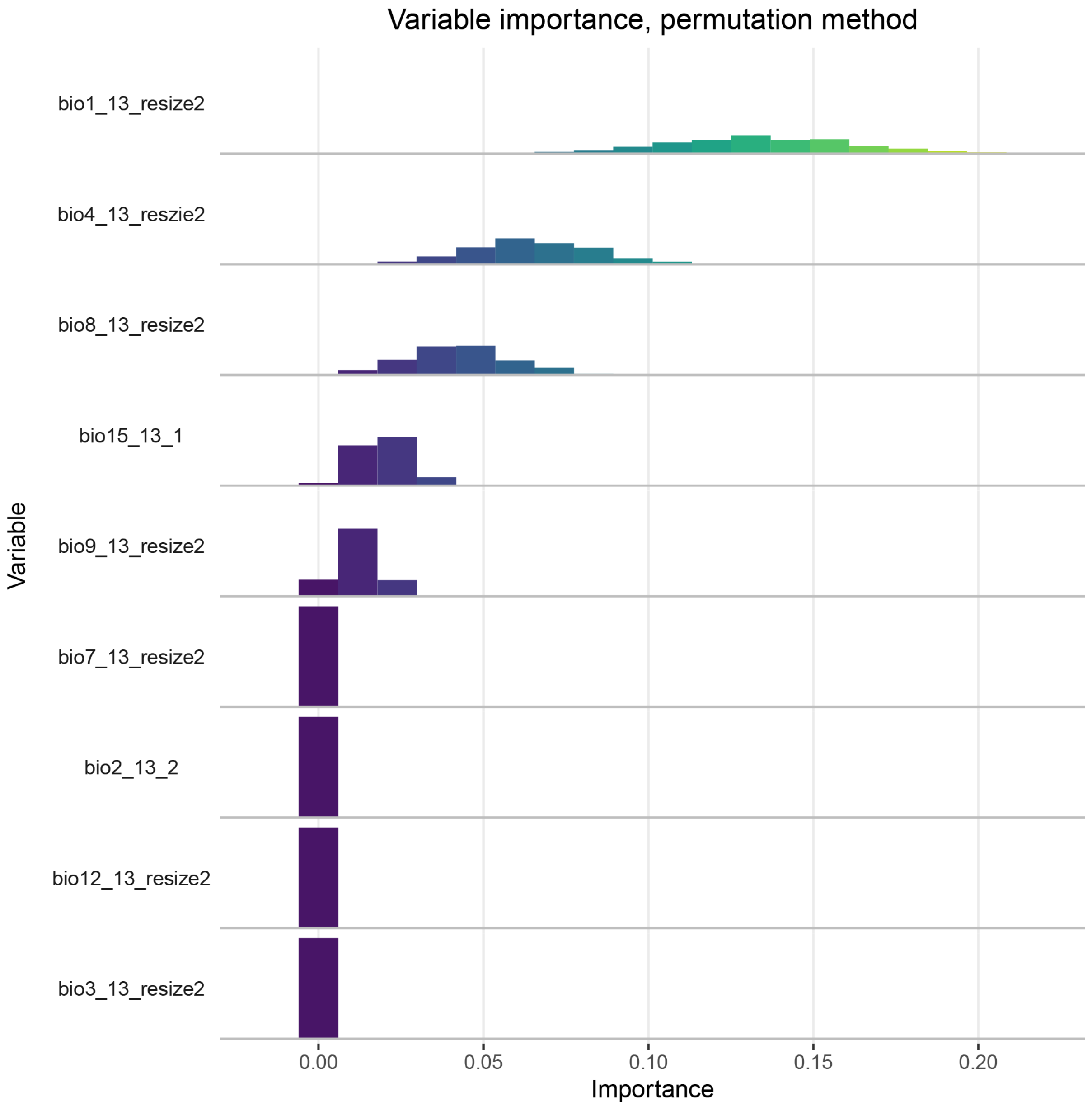

Figure S12: Variable importance plot for *P. clarkii* Maxent niche model using all variables.

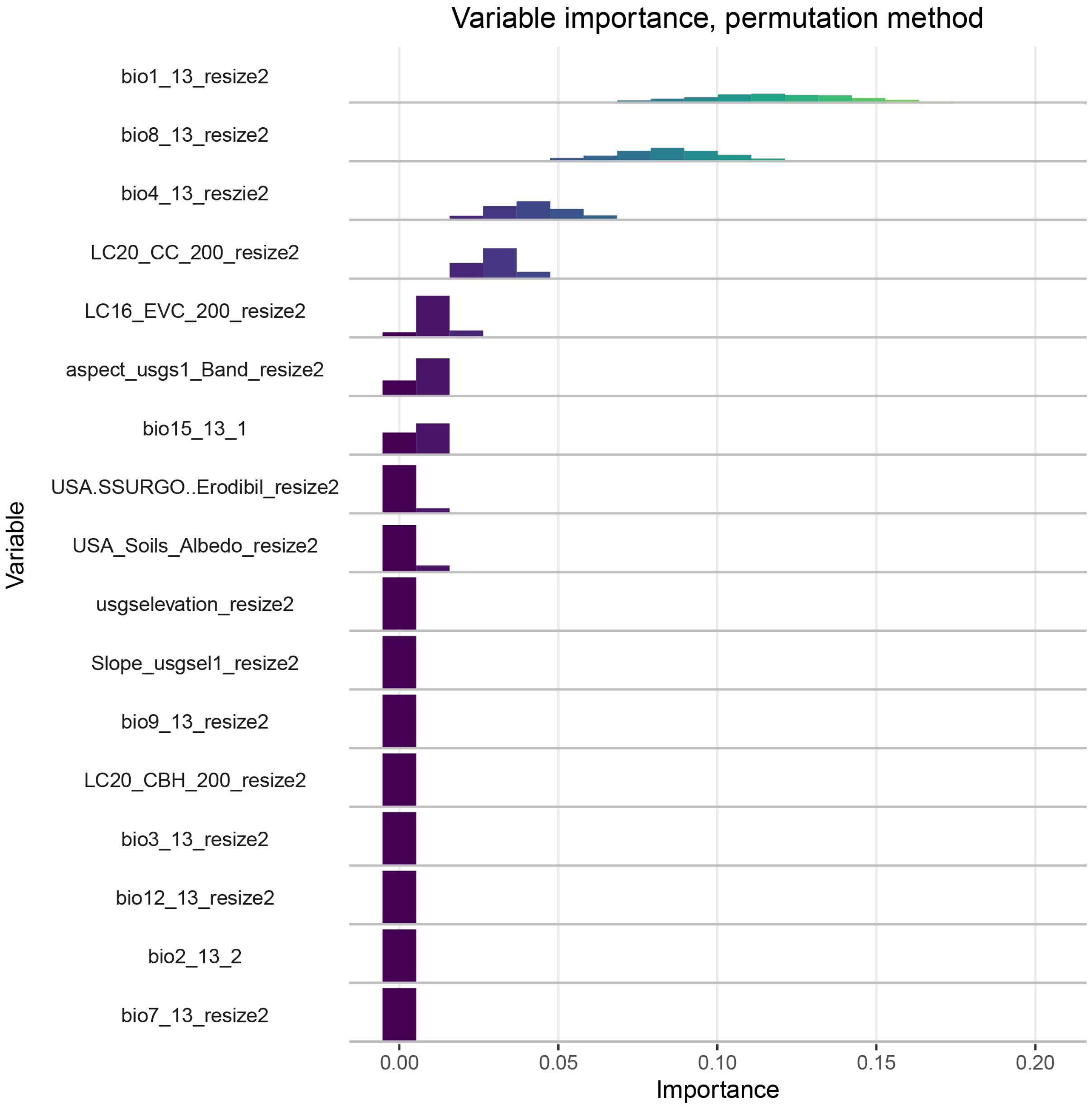

Figure S13: Variable importance plot for *P. nantahala* Maxent niche model using biotic, geological, and geographic variables.

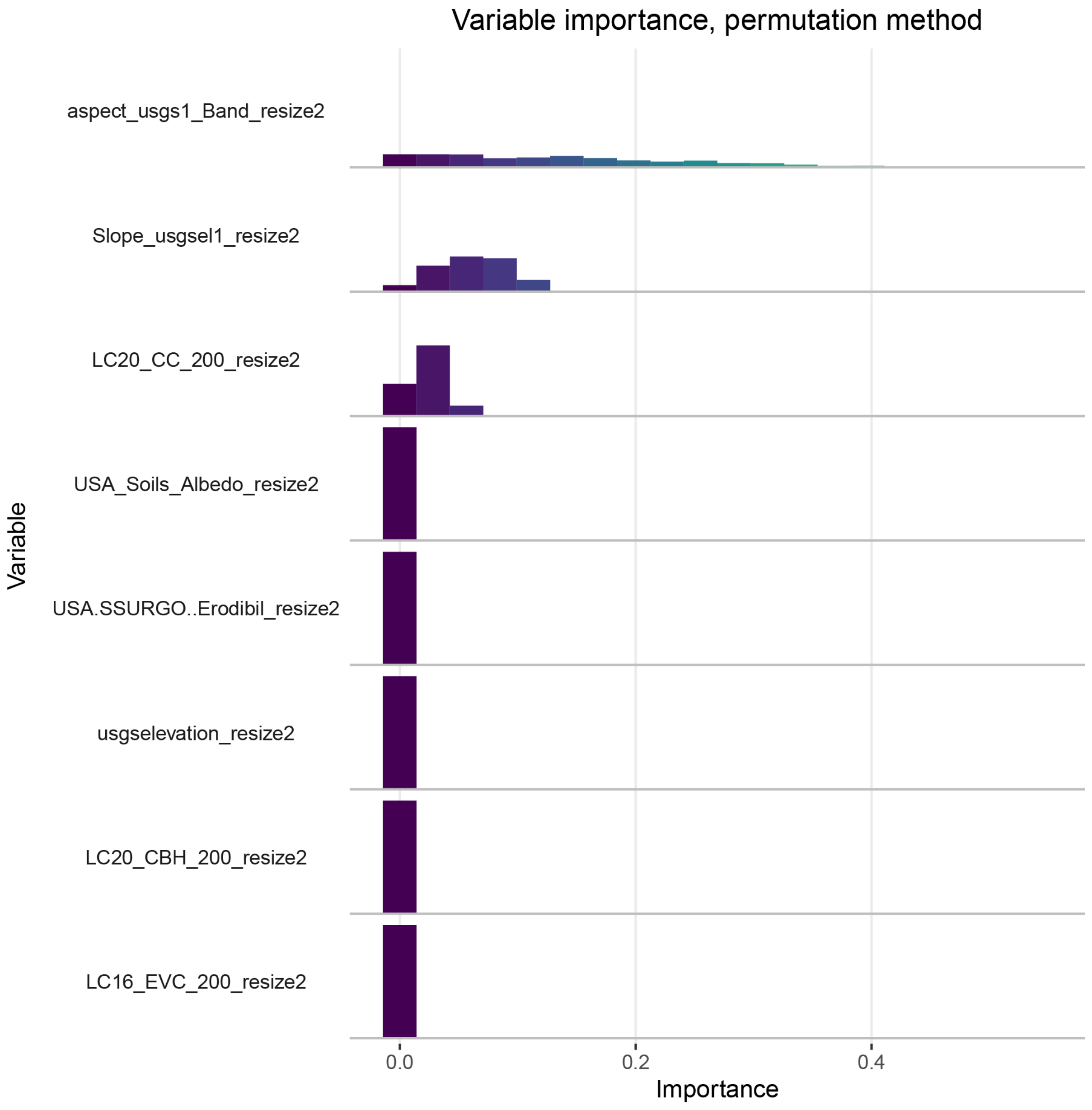

Figure S14: Variable importance plot for *P. nantahala* Maxent niche model using only bioclimatic variables.

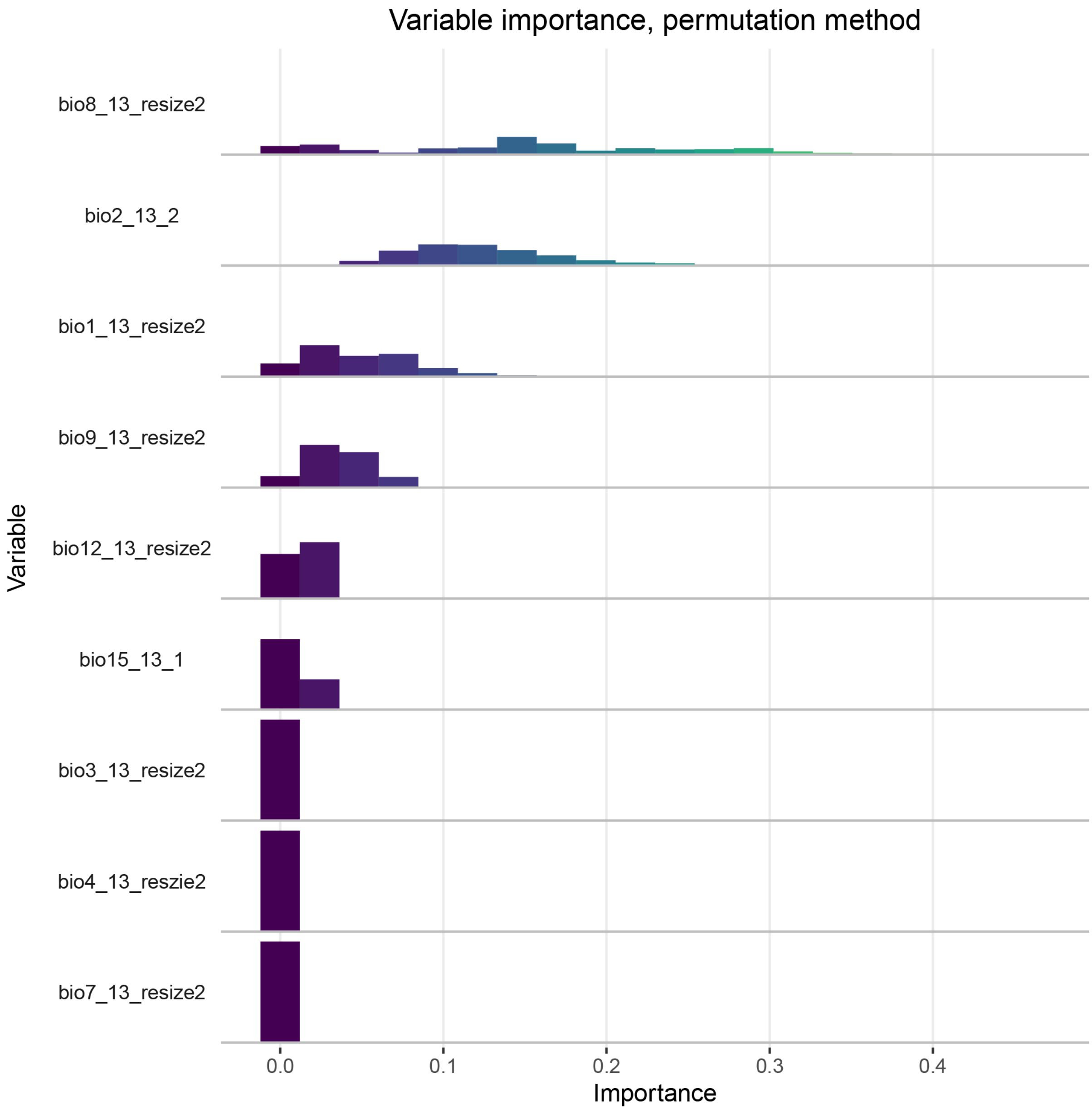

Figure S15: Variable importance plot for *P. nantahala* Maxent niche model using all variables.

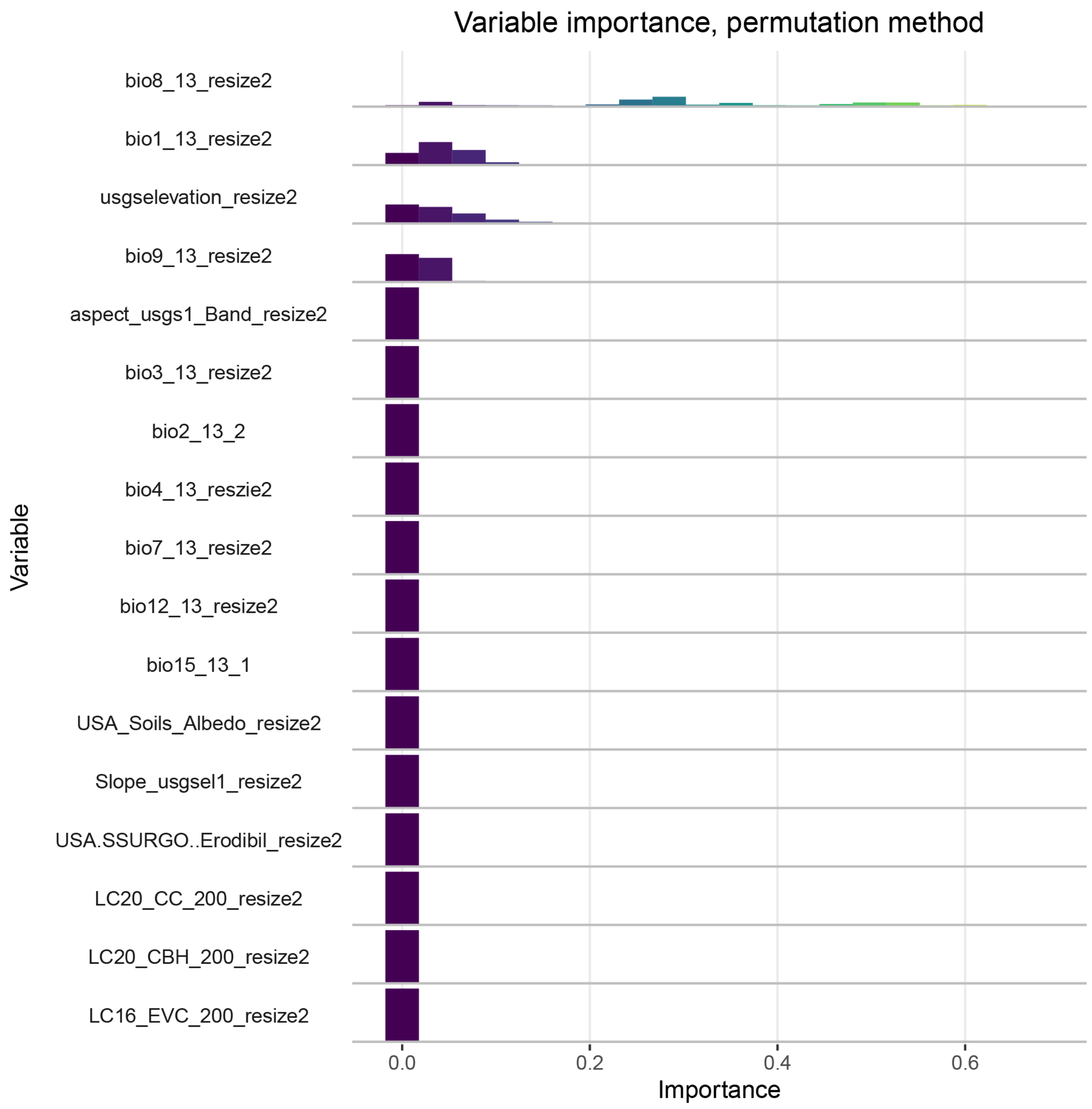

Supplement: Supplementary material 2 — Additional images [file zookeys-1158-091_article-94152__-s002.pdf]
